# Supplementary material for: Systems-wide analysis revealed shared and unique responses to moderate and acute high temperatures in the green alga Chlamydomonas reinhardtii
Source: Commun Biol. 2022 May 13;5:460. doi: 10.1038/s42003-022-03359-z (PMC9106746; doi:10.1038/s42003-022-03359-z)
Supplement: Supplementary file 2 — Supplementary Information [file 42003_2022_3359_MOESM2_ESM.pdf]

Systems-wide Analysis Revealed Shared and Unique Responses to Moderate and Acute High Temperatures in the Green Alga *Chlamydomonas reinhardtii*

Ningning Zhang[1,#], Erin M. Mattoon[1,2,#], Will McHargue[1], Benedikt Venn[3], David Zimmer[3], Kresti Pecani[4], Jooyeon Jeong[1], Cheyenne M. Anderson[1], Chen Chen[5], Jeffrey C. Berry[1], Ming Xia[1], Shin-Cheng Tzeng[1], Eric Becker[1], Leila Pazouki[1], Bradley Evans[1], Fred Cross[4], Jianlin Cheng[5], Kirk J. Czymmek[1], Michael Schroda[3], Timo Mühlhaus[3], Ru Zhang[1,*]

[#] Equal contribution

* Corresponding author

Category	35°C Unique Up	40°C Unique Up	Overlap Up	35°C Unique Down	40°C Unique Down	Overlap Down
H_0h	Amino acid metabolism, Mitochondrial electron transport	Heat stress, Protein processing, Carbon fixation	Heat stress, Protein folding/ degradation, RNA regulation, Lipid metabolism, Amino acid metabolism	DNA synthesis/ repair, Protein processing, RNA regulation		DNA synthesis, Protein processing, RNA regulation
H_0.5h		Heat stress, Protein processing	Protein folding/ degradation, Redox	Cell motility/division, DNA synthesis/ repair, Protein processing	Amino acid metabolism, Mitochondrial electron transport, Protein processing, RNA processing	Cell motility/division, Minor CHO metabolism, Nucleotide metabolism, Protein processing, RNA regulation
H_1h		Heat stress, Protein processing, Carbon fixation	Protein folding/ degradation, Rhodanase	Cell motility/ division, DNA synthesis/ repair, Protein processing	Fermentation, Mitochondrial electron transport, Photosynthesis, TCA cycle	Cell motility/ division, DNA synthesis, Minor CHO metabolism, Nucleotide metabolism, Protein processing, RNA regulation
H_2h		Protein processing, Starch synthesis, Carbon fixation, Amino acid metabolism	LHCII, Rhodonase	Cell motility/ division, DNA synthesis/ repair, Protein processing	Amino acid metabolism, Mitochondrial electron transport, Protein processing, RNA processing, TCA cycle, Transport	Cell motility/division, Minor CHO metabolism, Nucleotide metabolism, Protein processing, RNA regulation
H_4h		Carbon fixation, DNA synthesis/repair, Amino acid metabolism	LHCII	Cell motility/ division, DNA synthesis/ repair	Amino acid metabolism, riboflavin, Nucleotide metabolism, Protein processing, RNA processing, Transport	Cell motility/ division, DNA synthesis/ repair, Nucleotide metabolism, Protein processing, Photosynthesis, RNA regulation
H_8h	DNA synthesis/ chromatin structure, phosphogluconate dehydrogenase	Amino acid metabolism	Miscellaneous	Cell motility/ division	Amino acid metabolism, Cell motility/ division, DNA synthesis, Nucleotide metabolism, Protein processing, TCA cycle	Cell motility/ division, Nucleotide metabolism, Protein processing, Photosynthesis, RNA regulation
H_16h		Starch synthesis		Protein processing, S-assimilation, biodegradation of xenobiotics	Cell wall proteins, cell motility/ division, DNA synthesis, Nucleotide metabolism, Protein processing, TCA cycle	Protein processing, Redox, RNA regulation
H_24h	Cytochrome P450, RNA regulation	Protein processing	Secondary metabolism	RNA regulation	Cell wall proteins, Cell motility/ division, Nucleotide metabolism, Protein processing, Photosynthesis	Cell motility/ division, DNA synthesis, Nucleotide metabolism,
R_0h	Protein modification	Protein processing, Amino acid metabolism		DNA synthesis/ repair	Cell wall proteins, Riboflavin, Protein processing, Photosynthesis	Cell motility/ division
R_2h	Purine synthesis, Protein modification, RNA regulation		Cell division/ organization, DNA synthesis/ repair, Protein folding/ degradation, RNA regulation, G-proteins	Starch synthesis, Carbon concentrating mechanism	Cell motility/ division, Protein processing, Photosynthesis, Tetrapyrrole synthesis	Cell motility/ division, Lipid metabolism, Starch synthesis, Protein processing, Photosynthesis, TCA cycle, Tetrapyrrole synthesis
R_4h		Protein processing, RNA regulation	Cell division/ organization, DNA synthesis/ repair, glucosyl/ glucoronyl transferases, Protein folding/ degradation, RNA regulation, G-proteins	Carbon concentrating mechanism	Biodegradation of xenobiotics, Cell wall proteins, Lipid metabolism, Protein processing, Photosynthesis, TCA cycle, Tetrapyrrole synthesis	Fermentation, Photosynthesis, Redox
R_8h		DNA synthesis/ repair, Protein processing, RNA regulation	DNA synthesis/ repair		Protein processing, Photosynthesis	Fermentation, Photo-synthesis, Redox
R_24h		DNA synthesis/repair			Fermen-tation, Photo-synthesis	

Supplementary Table 1: MapMan functional enrichment summary for genes uniquely or overlappingly differentially expressed in 35°C and 40°C by time point.

For each time point, genes identified as uniquely up/down-regulated or overlappingly up/down-regulated in 35°C and 40°C treatments were used for MapMan functional enrichment analysis. Significantly enriched MapMan terms (FDR < 0.05) were sorted into broad functional categories (see Supplementary Data 3 for detailed functional information). Broad functional categories with at least one significantly enriched MapMan term are displayed in this table. Time points during heat: H_0h, reach high temperature of 35°C or 40°C; H_0.5h, heat at 35°C or 40°C for 0.5 h, similar names for other time points during heat. Time points during recovery: R_0h, reach control temperature of 25°C for recovery after heat; R_2h, recovery at 25°C for 2 h, similar names for other time points during recovery.

Supplementary Table 2: Primers used for RT-qPCR.

Gene	Forward primers	Reverse primers
CBLP	GTCATCCACTGCCTGTGCTT	CCTTCTTGCTGGTGATGTTG
HSP90A	CGCCTACATGACCTCCAAGA	CACCTCCTCGTCAATGGACAG
HSP22A	CGCAGTCAACCGCATGATTA	CTGCAGCTCCACTTTGACG
HSF1	ATGTGCTCATGGTTGAGCTG	TTGATCATGGTCTGCTGCTT
HSF2	TCCTGCGCAAGACATATGAG	CAAACTCGCTAGGCTTCCAG
Lhca1	GTGAGCTGAAGCTGAAGGAG	TATCAGGAGATCATTTGCATGTGG
Trxf2	CTGGACAAGGACACGTTCTG	GCAGTTGACCTTGACGAACC
LHCBM4	ACTTTACTAGCAGAAATGGCGT	GGGTCGAGTTCTCAGAGTAGG
HAT4	GTACAGCACTCAGTCGTACAC	GTACTCCACCTTGTCCACCA
CAM8	CTTTCACGCTGTTCGACTCC	GGTCCTGCTCATTGTCATACTC

Supplementary Table 3: Formulas used to calculate photosynthetic parameters.

Description	Label	Units	Formula
Maximal chlorophyll fluorescence, in dark-adapted algal cells	F_m		
Maximal chlorophyll fluorescence, in light-adapted algal cells	F_m'		
Minimal chlorophyll fluorescence, in dark-adapted algal cells	F_o		
Minimal chlorophyll fluorescence, in light-adapted algal cells	F_o'		
Steady state fluorescence in light-adapted algal cells	F_s		
PSII efficiency	ΦPSII		$\text{For dark}-\text{adapted algal cells}, \Phi\text{PSII} = 1 - \frac{F_o}{F_m}$; $\text{For light}-\text{adapted algal cells}, \Phi\text{PSII} = 1 - \frac{F_s}{F_m'}$
PSII relative antenna size	PSII%		PSII%= PSII peak / (PSII peak + PSI peak) PSII and PSI peak were measured by 77 K chlorophyll fluorescence; both peaks were normalized to PSII spectral maximum at 686 nm
Linear electron flow	LEF	μmol m^{-2} s^{-1}	$\text{LEF} = (\Phi\text{PSII})(\text{PSII\%})(\text{Qabs}_{\text{fs}})$ $Qabs_{fs}$: fraction of absorbed light, assuming 0.8
Fraction of open PSII centers	q_L		$q_L = q_P * \frac{F_o'}{F_s}$ = $(F_q'/F_v') \times (F_o'/F_s)$ = $(F_m' - F_s)/(F_m' - F_o')] \times (F_o'/F_s)$
Plastoquinone redox status	Q_A		$Q_A = 1 - q_L$
Non-photochemical quenching	NPQ		$\text{NPQ} = \frac{(F_m - F_m')}{F_m'}$

Supplementary Figures below

Supplementary Fig. 1

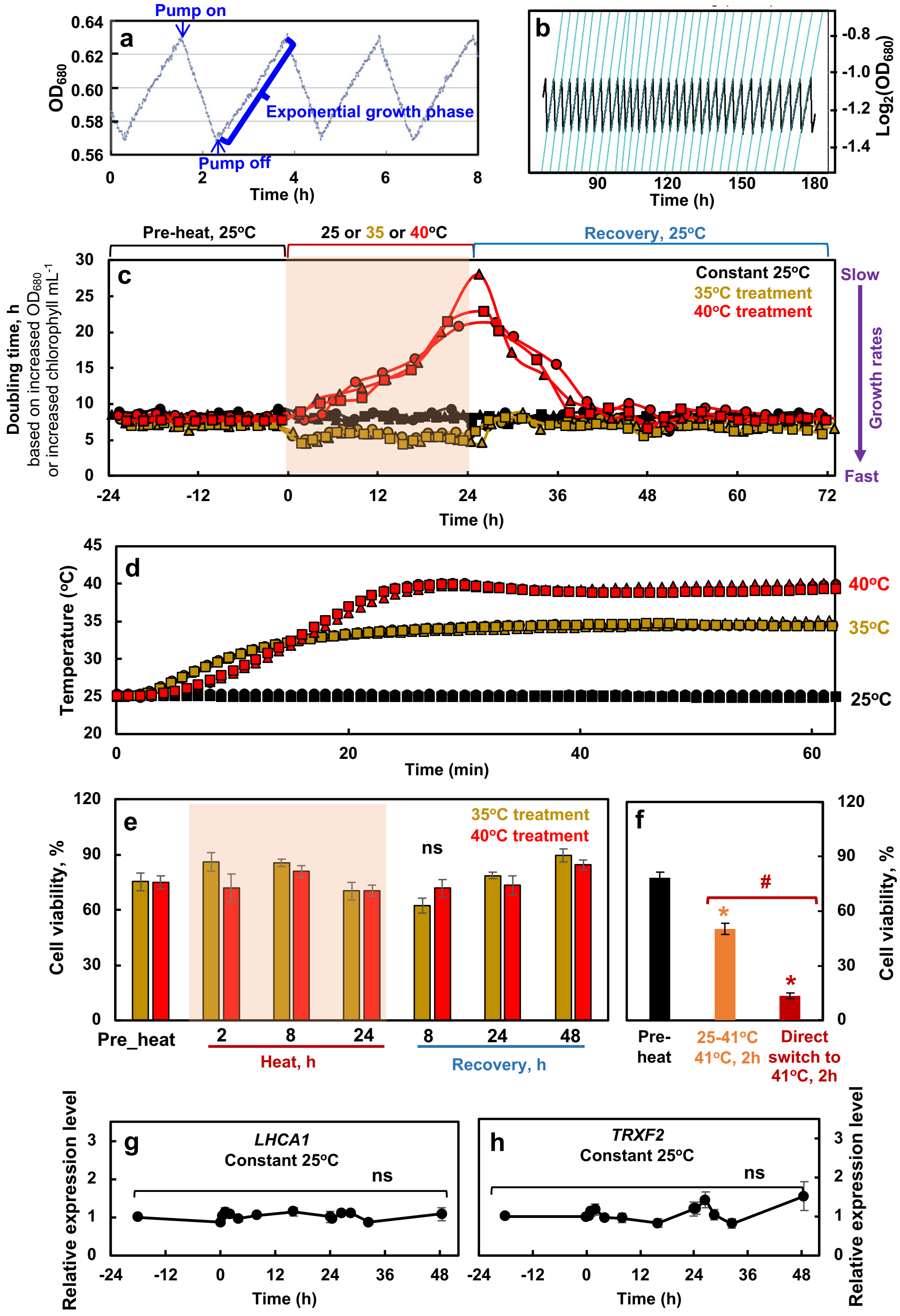

Supplementary Fig. 1: Algal cultures were grown in photobioreactors (PBRs) under well-controlled conditions with turbidostatic mode for different temperature treatments. (a) PBR cultures were grown with air level CO_2 in Tris-acetate-phosphate (TAP) medium under constant light (100 µmol photons m^{-2} s^{-1} with equal amount of blue and red light) turbidostatically within a small range of OD_{680} which monitors chlorophyll content. When PBR cultures grew to the set maximum OD_{680}, pumps turned on to add fresh medium and dilute the cultures to the set minimal OD_{680}, then pumps turned off to allow for exponential growth to the maximum set OD_{680}. (**b**) The doubling time of the PBR cultures were calculated by fitting the OD_{680} curves during exponential growth. (**c**) Doubling time of the PBR cultures before, during and after treatment at 35°C, 40°C, or constant 25°C. Doubling time is inverse of relative growth rates and smaller doubling time represents faster growth based on the rate of chlorophyll increase. Three independent biological replicates are plotted for each treatment. Constant 25°C served as controls which showed steady growth without heat treatment. **(d)** PBR heating profiles. PBR temperatures changed from 25°C to 35°C or 40°C gradually within 30 min. Three independent biological replicates are plotted for each temperature treatment. **(e)** Heat treatment in PBRs at 35°C or 40°C up to 24 h did not affect cell viability. Algal cells with different heat treatments were diluted and spotted on TAP plates, grown under 150 µmol photons m^{-2} s^{-1} white LED light, 25°C for 44 h before microscopic imaging. Colony numbers were quantified using ImageJ. Cell viability was calculated as the number of colonies on plates divided by the number of cells spotted. Values are mean ± SE, n = 3 biological replicates. Statistical analyses were performed with two-tailed t-test assuming unequal variance by comparing different time points with the pre-heat samples. No significance (ns) among different time points ($p>0.05$). (**f**) Heating speed affected algal cell viability and direct heating at 41°C in water bath significantly reduced algal cell viability. Algal cultures were harvested from PBRs before heat treatment (pre-heat, black bars), or incubated in a water bath which was heated from 25°C to 41°C gradually in 25 min then kept at 41°C for 2 h (orange bars), or directly heated in a water bath which was pre-heated to 41°C (sharp temperature switch) then kept at 41°C for 2 h (red bars). Cell viability was quantified as in (**e**). Values are mean ± SE, n = 3 biological replicates. Statistical analyses were performed using two-tailed t-tests assuming unequal variance by comparing treated

samples with pre-heat (*, p<0.05, the colors of asterisks match the treatment conditions) or between the two heating methods (#, p<0.05). (**g, h**) The circadian regulated genes *LHCA1* and *TRXF2* had constant expression levels without heat treatments. The relative expressions were calculated from RT-qPCR by normalizing to the reference gene *CBLP* and pre-heat-stress level. Mean ± SE, *n* = 3 biological replicates. Statistical analyses were performed with two-tailed t-test assuming unequal variance by comparing different time points with the first time point. No significance (ns) among different time points (p>0.05). (**c**, **e**) Red shaded area depicts the duration of high temperatures.

Supplementary Fig. 2

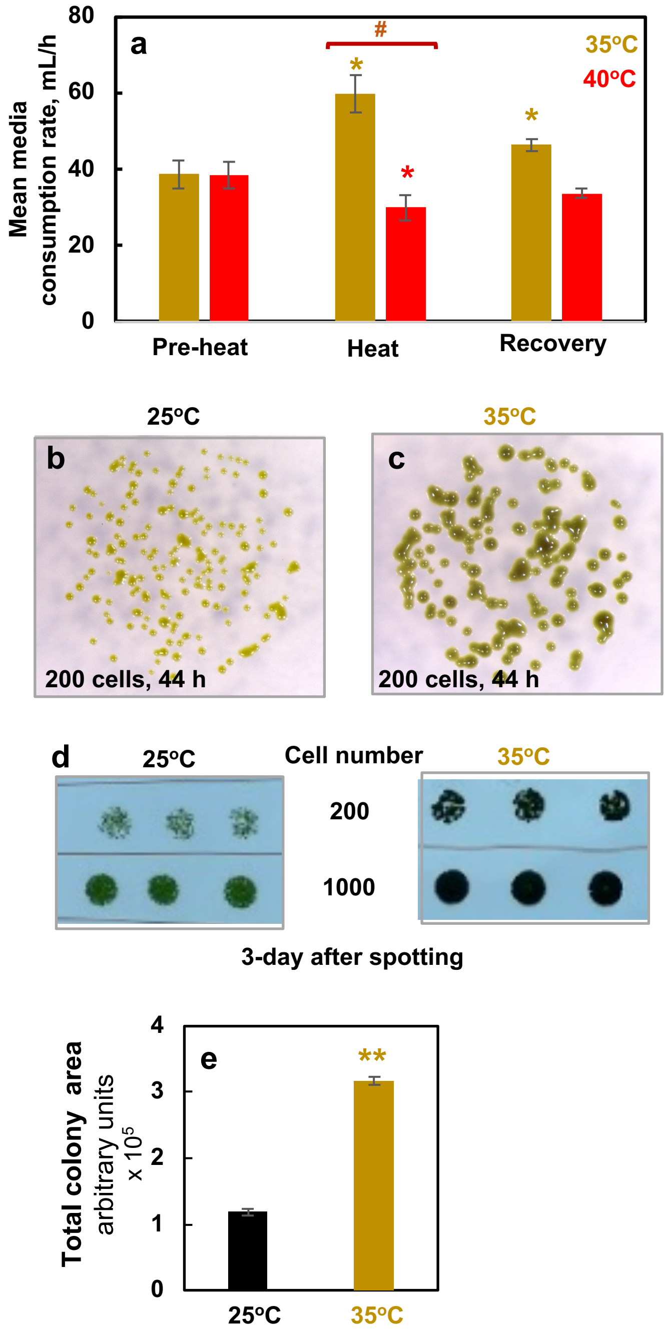

Supplementary Fig. 2. Moderate high temperature at 35°C increased algal growth rates. (**a**) PBR mean media consumption before heat, at the end of 24-h heat of 35°C or 40°C, and at the end of the recovery at 25°C, total media consumption volume divided by time. Cell growth induced culture dilution through turbidostatic control and consumed medium. Mean ± SE, n = 4. (**b-e**) The increased growth rates under 35°C were confirmed

by spotting tests on plates. Algal cells harvested from PBRs at 25°C without heat treatments were diluted and spotted on TAP plates, grown under 150 µmol photons m^{-2} s^{-1} white LED light, in incubators of 25°C or 35°C for 44 h (**b, c**) or 3 days (**d**) before imaging. (**b, c, d**) One of the three biological replicates was shown. (**e**) Algal spots with 200 cells were imaged after 44-h growth and colony areas were quantified using ImageJ. Values are mean ± SE, *n* = 3 biological replicates. (**a**, **e**) Statistical analyses were performed with two-tailed t-test assuming unequal variance by comparing treated conditions with the pre-heat (**a**) or the 25°C conditions (**e**) (*, $p<0.05$; **, $p<0.01$; the colors of asterisks match treatment conditions) or between 35°C and 40°C heat treatment (#, $p<0.05$).

Supplementary Fig. 3

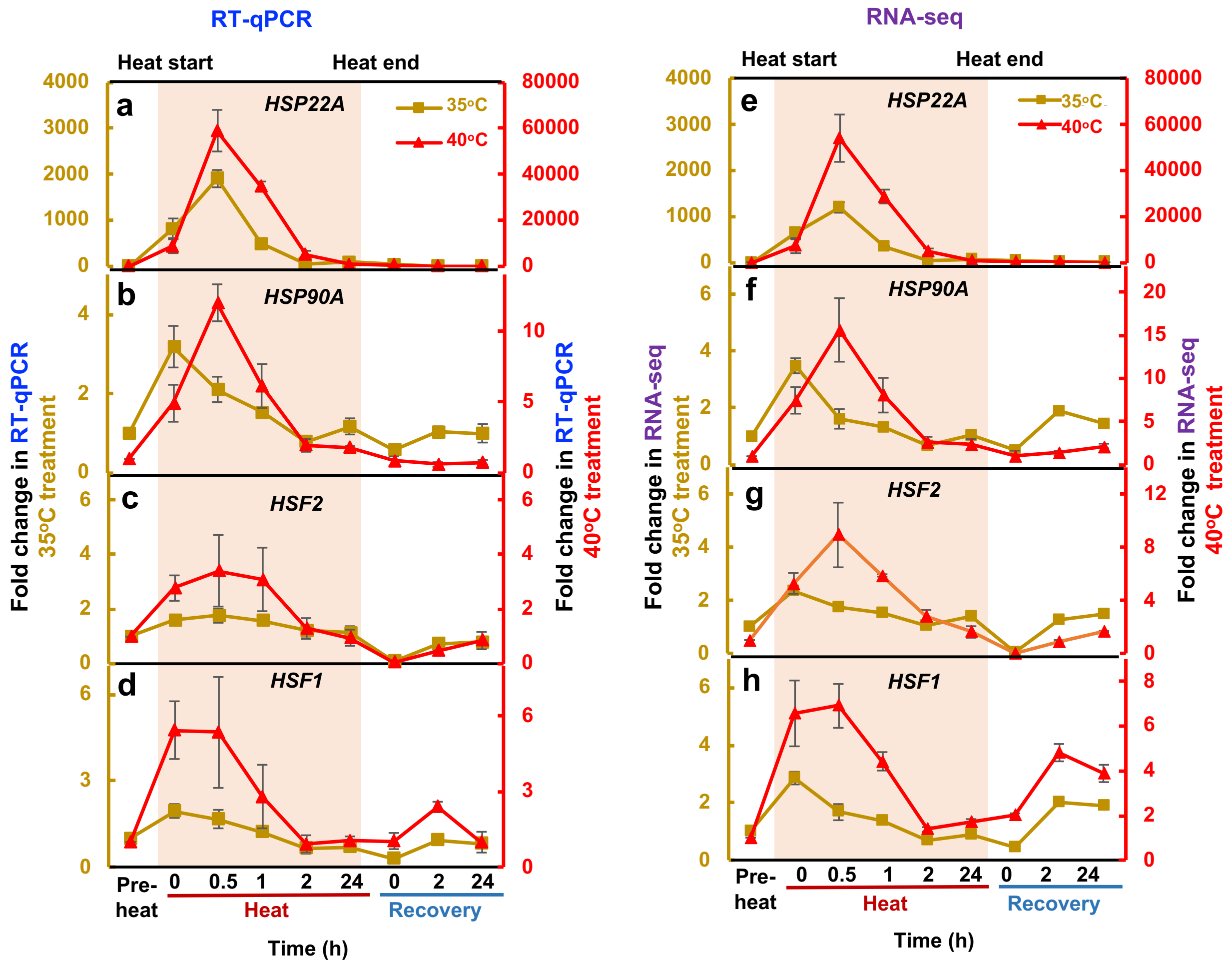

Supplementary Fig. 3. RT-qPCR analysis was consistent with RNA-seq results. Transcript fold-changes of two heat stress marker genes (*HSP22A*, *HSP90A*) and two heat shock transcription factors (*HSF1* and *HSF2*) were calculated based on RT-qPCR (**a-d**) or RNA-seq (**e-h**) results. Different y scales were used for samples with 35°C (left) or 40°C (right) treatments. Red shaded area depicts the duration of high temperature. For RT-qPCR results, the fold-changes were calculated by normalizing the relative expression values at different time points with different treatments to the reference gene *CBLP* and the pre-heat time point. For RNA-seq results, the fold-changes were calculated based on Transcripts Per Million (TPM) normalized RNA-seq read counts. Values are mean ± SE, *n* = 3 biological replicates.

Supplementary Fig. 4

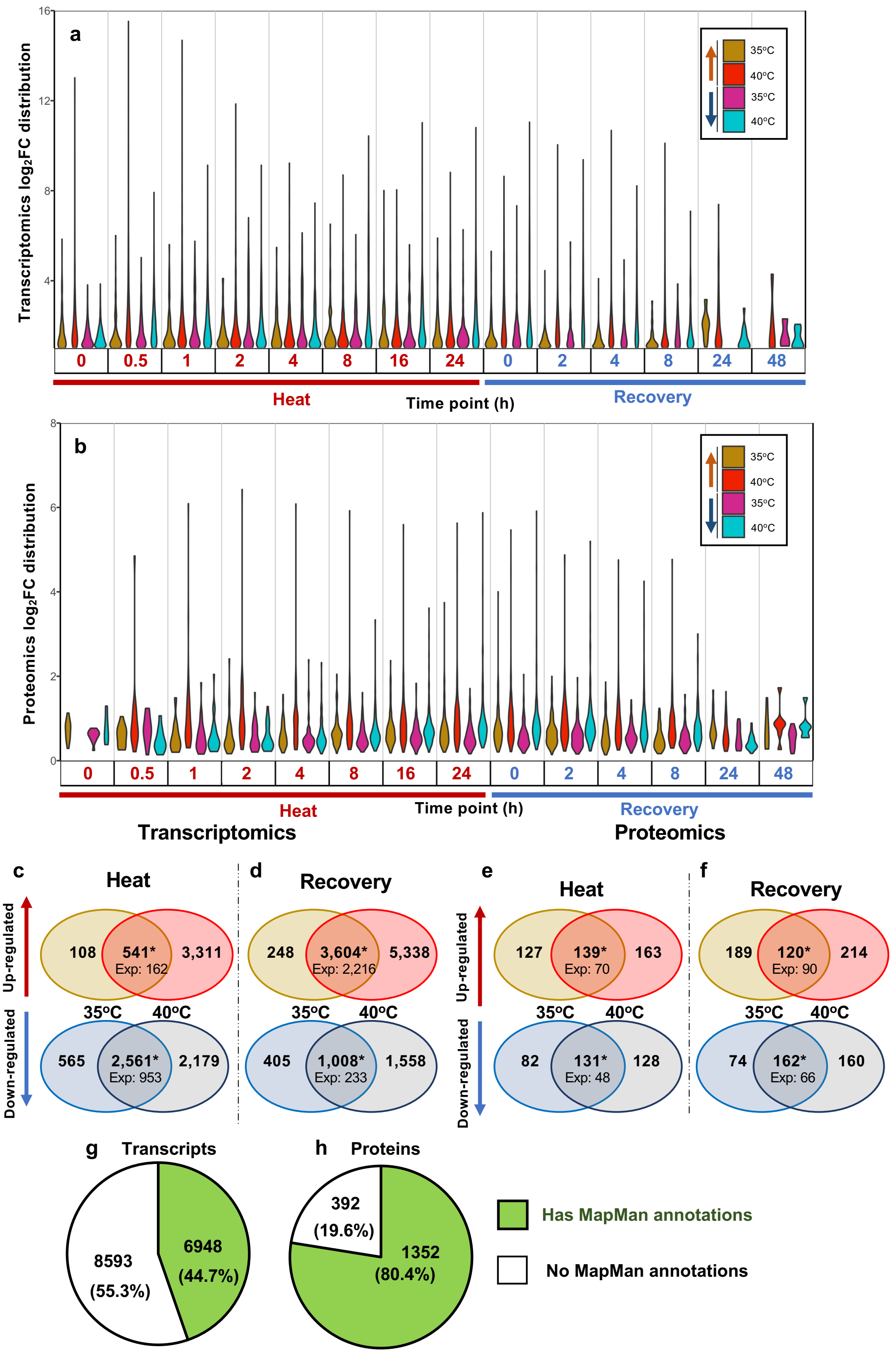

Supplementary Fig. 4: Transcriptomic and proteomic analyses revealed shared and unique regulation of transcripts and proteins during and after heat treatments of 35°C or 40°C. (a, b) Log_2(fold-change, FC) distribution of Differentially Expressed Genes (DEGs) and Differentially Accumulated Proteins (DAPs) at different time points, respectively. For each time point, the first two violins represent up-regulated transcripts/proteins, and the last two violins represent down-regulated transcripts/proteins. For down-regulated transcripts/proteins, the inverse of the log_2FC was displayed. The width of the violins is proportional to the fraction of transcripts/proteins at a certain fold-change value out of the total DEGs/DAPs at a given time point. Time points during heat: 0 h, reach high temperature of 35°C or 40°C; 0.5 h, heat at 35°C or 40°C for 0.5 h, similar names for other time points during heat. Time points during recovery: 0 h, reach control temperature of 25°C for recovery after heat; 2 h, recovery at 25°C for 2 h, similar names for other time points during recovery. (**c-f**) Venn diagrams of transcripts (**c**, **d**) and proteins (**e, f**) differentially expressed in at least one time point during heat treatment (**c, e**) or recovery (**d, f**). For each panel: top, up-regulated transcripts/proteins; bottom: down-regulated transcripts/proteins. Only transcripts and proteins identified in both 35°C and 40°C treatment groups were used for this analysis. Expected values are the number of transcripts/proteins expected to have overlapping differential expression between the 35°C and 40°C treatment groups based on random chance (Fisher's exact test, *: $p< 1.29 \times 10^{-226}$). **(g, h)** Pie chart of transcripts **(g)** and proteins **(h)** in our analyses that have at least one MapMan annotation (green) versus no MapMan annotations (white).

Supplementary Fig. 5

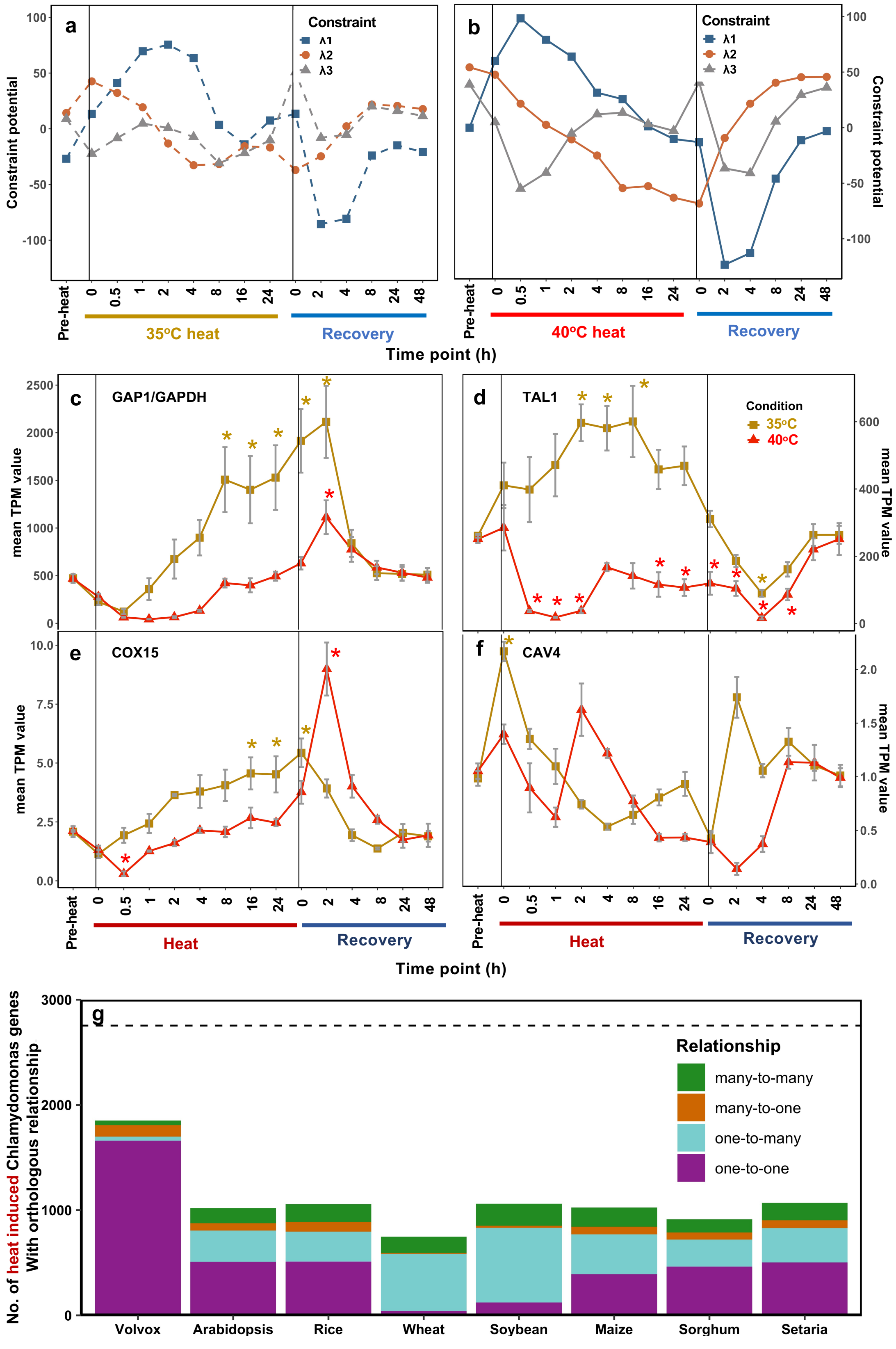

Supplementary Fig. 5: Global transcription patterns were similar between 35°C and 40°C treatments, but detailed analysis revealed uniquely differentially expressed genes in the 35°C treatment. Time course of the three major constraint potentials (λ1-3) derived from surprisal analysis for 35°C (**a**) and 40°C (**b**) experiments, respectively. The constraint potentials indicate the most important transcriptional patterns during the time course. **(c-f)** Mean transcript per million (TPM) read counts at each time point for select genes that were uniquely up-regulated during 35°C (brown) but not 40°C (red) heat treatment period. Values are mean ± SE, *n* = 3 biological replicates, asterisks indicate significance in differential expression modelling. (**c**) GAP1/GAPDH: Cre12.g485150, Glyceraldehyde 3-phosphate dehydrogenase, involved in gluconeogenesis, glycolysis, and Calvin-Benson Cycle; (**d**) TAL1: Cre01.g032650, transaldolase, involved in the pentose phosphate pathway, which acts upstream of the glycolytic and gluconeogenic pathways; (**e**) COX15: Cre02.g082700, encoding mitochondrial cytochrome c oxidase assembly factor; (**f**) CAV4: Cre11.g467528, encoding a putative voltage-gated calcium channel. Vertical black lines indicate the start and end of heat treatments. Time points were labeled at the bottom. (**a-f**) Pre-heat, before heat treatments. Time points during heat: 0 h, reach high temperature of 35°C or 40°C; 0.5 h, heat at 35°C or 40°C for 0.5 h, similar names for other time points during heat. Time points during recovery: 0 h, reach control temperature of 25°C for recovery after heat; 2 h, recovery at 25°C for 2 h, similar names for other time points during recovery. (**G**) Conservation of Chlamydomonas heat induced genes (HIGs), which are up-regulated in at least one time point of 35°C or 40°C high temperature period, with select land plant species. Orthologous relationships were determined using JGI InParanoid data. Dashed horizontal line indicates the number of Chlamydomonas HIGs that were present in the JGI InParanoid data (2754 genes out of 3960 HIGs total). Abbreviated species names on x-axis correspond with the following JGI genomes: Volvox (*Volvox carteri*, Vcarteri_v2.1), Arabidopsis (*Arabidopsis thaliana*, Athaliana_TAIR10), Rice (*Oryza sativa*, Osativa_v7.0), Wheat (*Triticum aestivum*, Taestivum_v2.2), Soybean (*Glycine max*, Gmax_Wm82.a2.v1), Maize (*Zea mays*, Zmays_RefGen_V4), Sorghum (*Sorghum bicolor*, Sbicolor_v3.1.1), Setaria (*Setaria viridis*, Sviridis_v2.1).

Supplementary Fig. 6

a Overlapping up-regulated genes between 35°C and 40°C

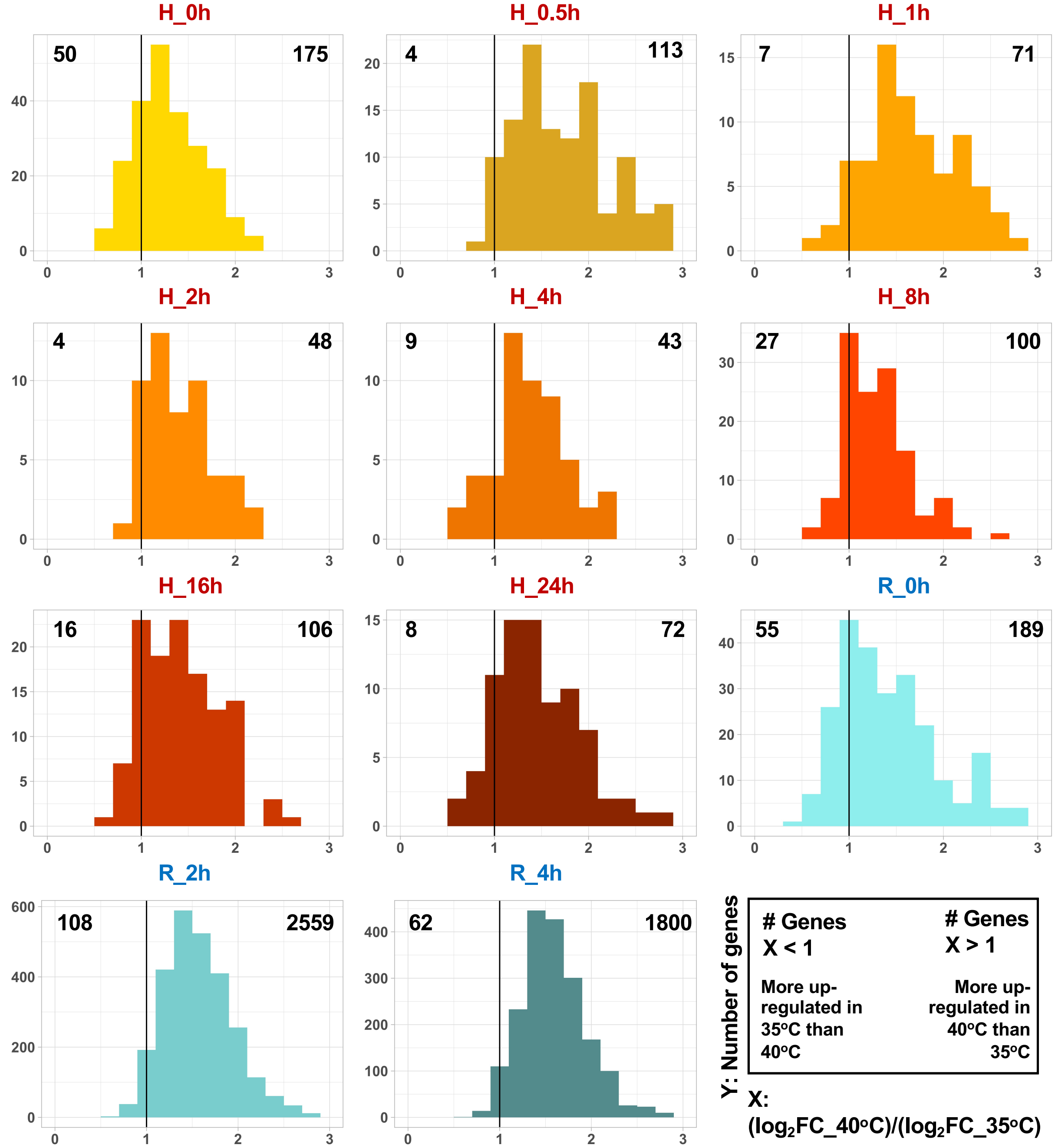

Supplementary Fig. 6

b Overlapping down-regulated genes between 35°C and 40°C

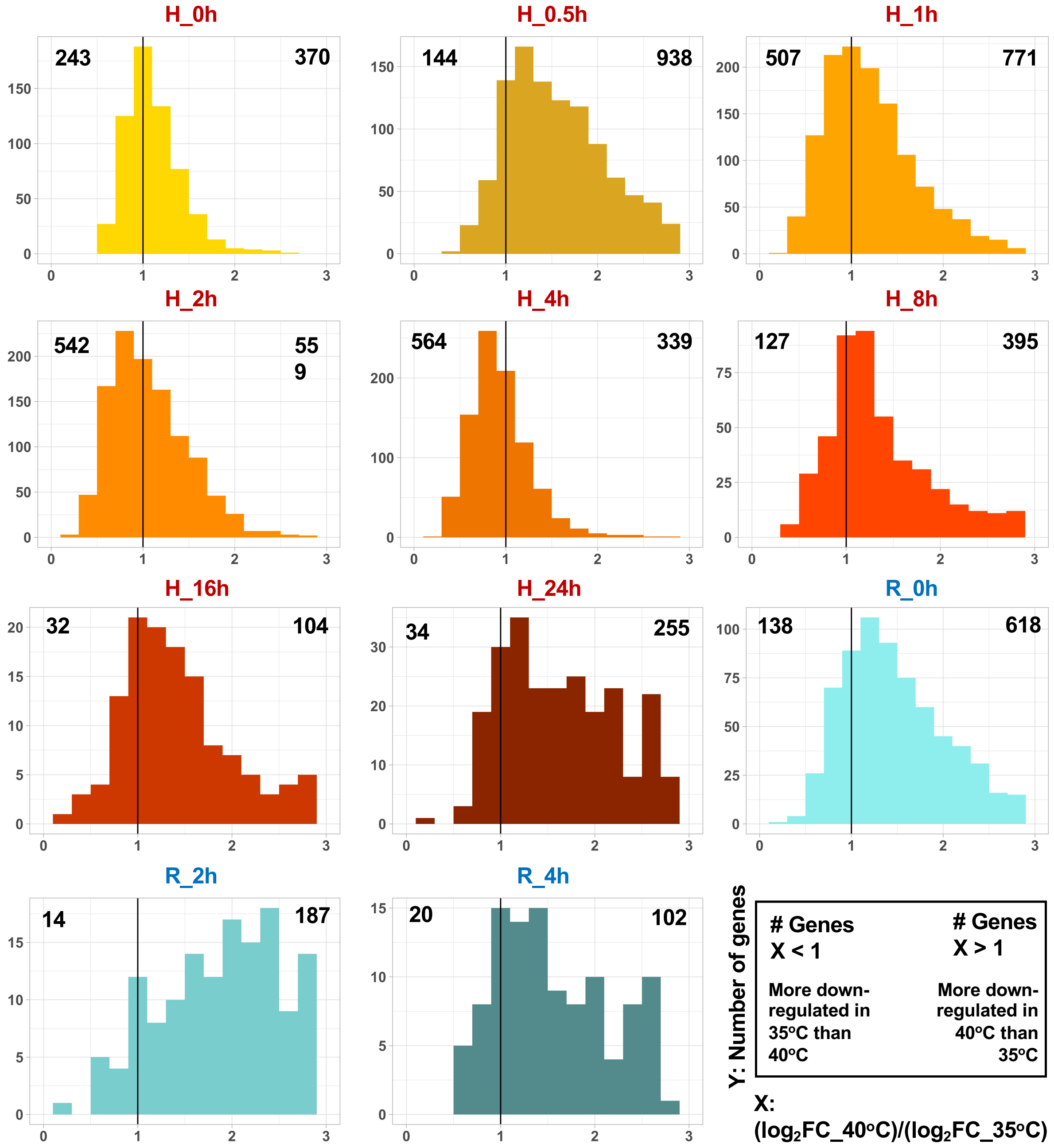

Supplementary Fig. 6: Some overlapping DEGs between 35ºC and 40ºC were more differentially regulated with 35ºC than 40ºC treatment. DEGs, differentially expressed genes. FC, fold-change. Histograms of log_2(FC in 40ºC)/log_2(FC in 35ºC) for overlapping up-regulated (**a**) and down-regulated (**b**) genes between 35ºC and 40ºC are displayed for each time point. Very few overlapping DEGs between 35ºC and 40ºC were identified at

8, 24, and 48 h of recovery, which were thus omitted. Black vertical lines indicate equal differential expression between 35°C and 40°C treatments. Bars to the left of the black line indicate genes more differentially expressed in the 35ºC treatment group while bars to the right of the black line indicate genes more differentially expressed in the 40ºC treatment group. Numbers in the top left and right corners of each histogram represent the number of genes with higher fold change values in 35°C or 40ºC, respectively. H_0h, reach high temperature of 35°C or 40°C. H_0.5h, heat at 35°C or 40°C for 0.5 h, similar names for other time points during heat. R_0h, reach control temperature of 25°C for recovery after heat. R_2h, recovery at 25°C for 2 h, similar names for other time points during recovery.

Supplementary Fig. 7

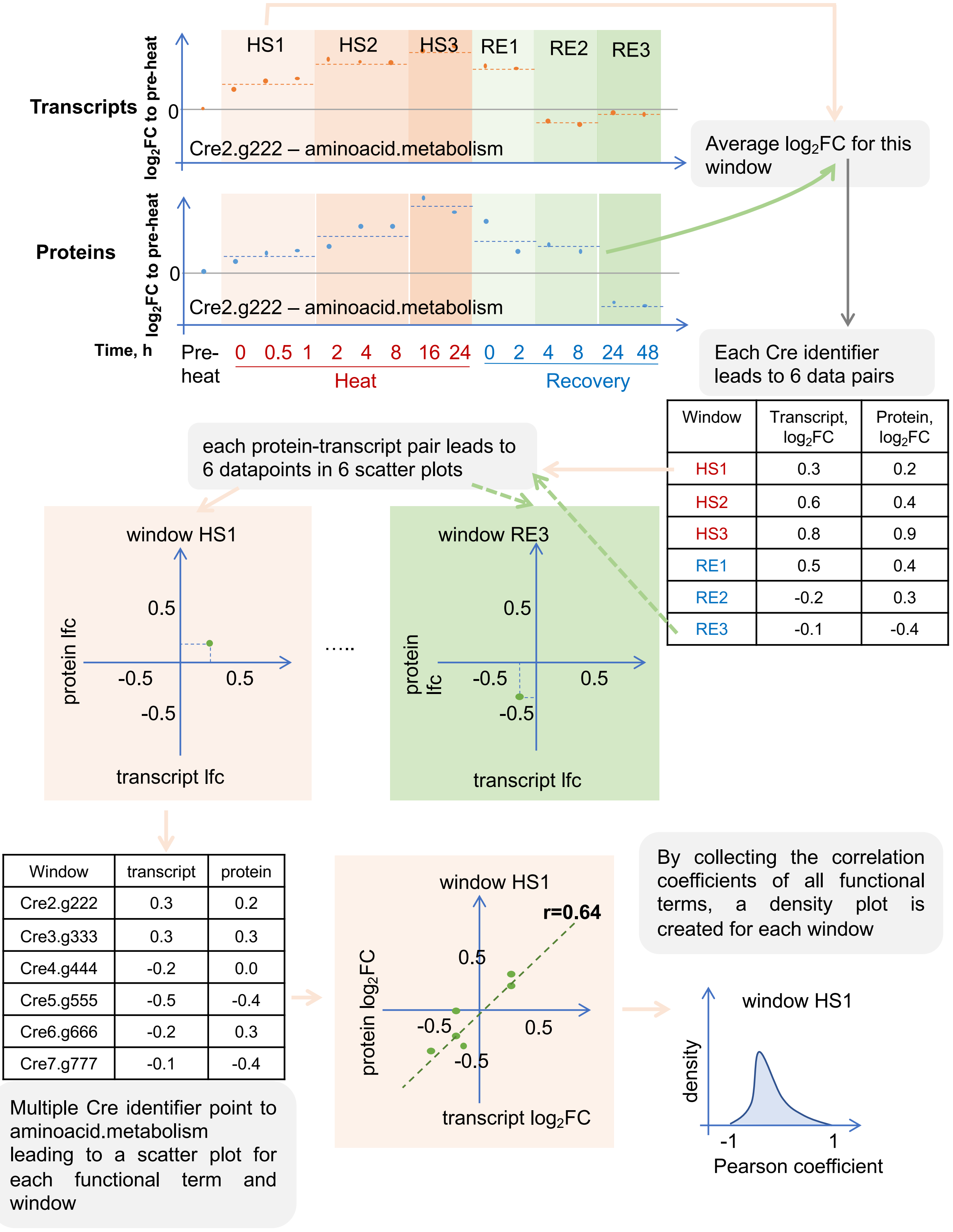

Supplementary Fig. 7: The fold-change correlation between transcripts and proteins were investigated. Transcript and protein correlation analysis by using Cre2.g222 (hypothetical gene ID as Cre identifier) of functional term aminoacid.metabolism as an example. Log_2(fold-changes, FC) of transcript reads and protein abundance are calculated in respect the pre-heat sample. The high temperature period (HS) as well as the recovery period (RE) are split into three windows each (HS1-3 and RE1-3). HS1-3 windows: 0-1 h, 2-8 h, 16-24 h during the heat period; RE1-3 windows, 0-2 h, 4-8 h, 24-48 h during the recovery period after heat treatment. Every identifier that has transcripts as well as proteins associated to it, results in a transcript-protein fold-change pair for each window. The average Log_2FC is determined for each transcript-protein pair in each window. By collecting all Cre identifiers that are associated with aminoacid.metabolism, a scatter plot of transcript-protein fold-change pairs is generated and the Pearson correlation coefficient is calculated. By repeating the workflow for all functional terms for each window, a density plot for each of the six windows is created to describe the overall correlation of transcript reads and protein abundance for that window.

Supplementary Fig. 8

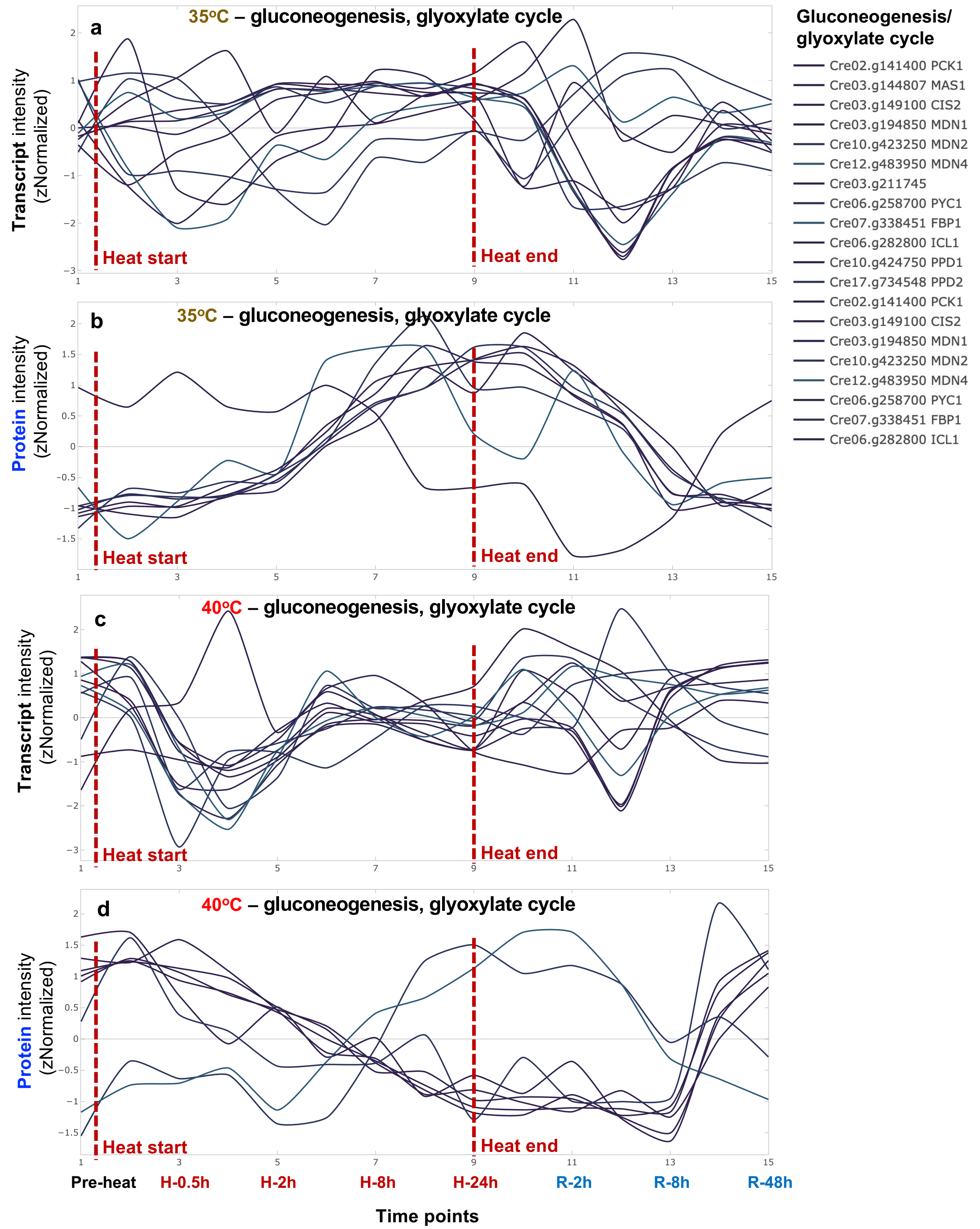

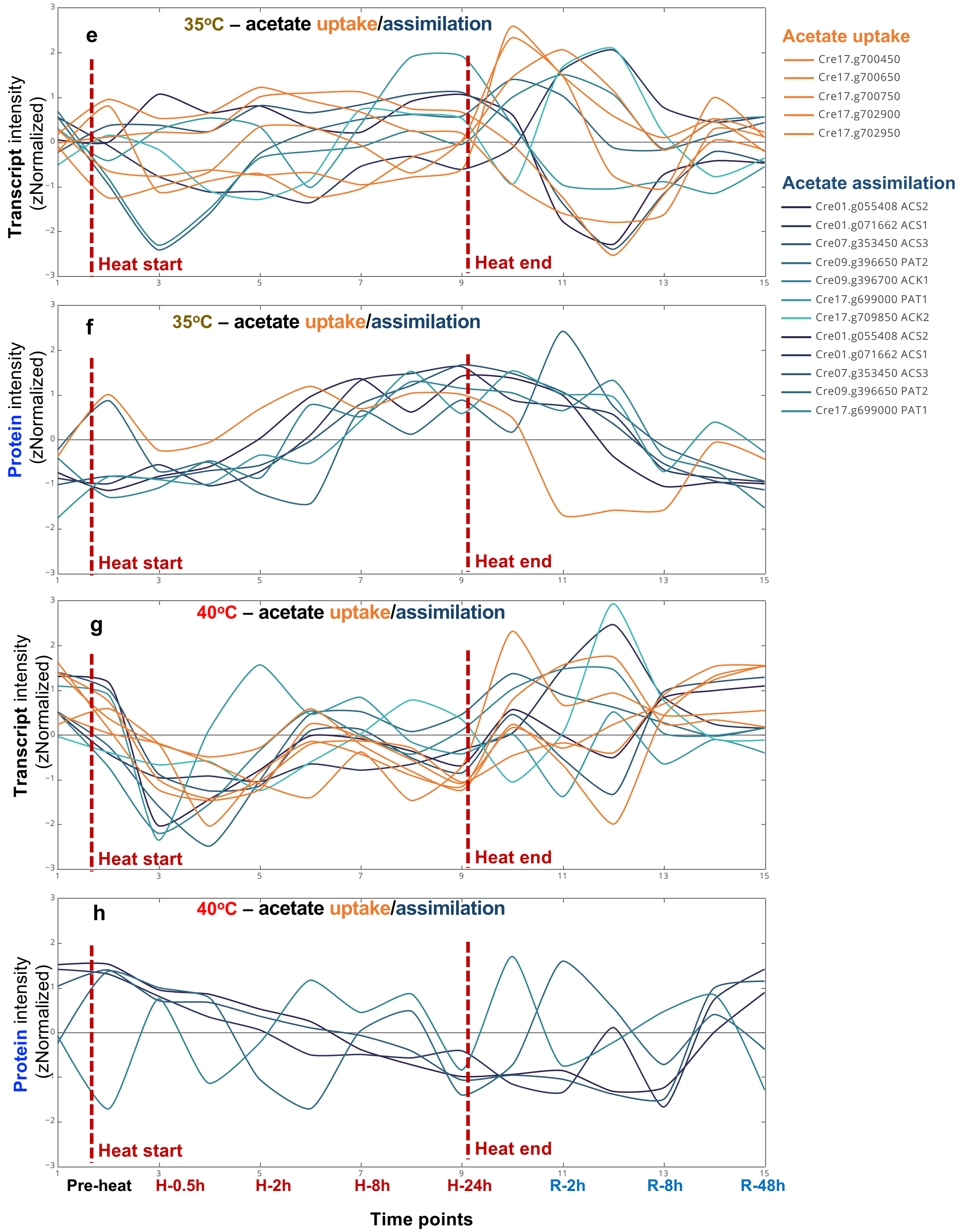

Supplementary Fig. 8. The kinetics of transcripts and proteins suggest gluconeogenesis/glyoxylate cycle and acetate uptake/assimilation increased

during 35°C but decreased during 40°C heat. Transcript (**a**, **c, e, g**) and protein (**b**, **d, f, h**) signals related to the gluconeogenesis/glyoxylate cycle and acetate uptake/assimilation were standardized to z scores (standardized to zero mean and unit variance) and are plotted against equally spaced time point increments. The red dashed lines indicate the start and end time of heat treatment for 35°C (**a**, **b, e, f**) and 40°C (**c**, **d, g, h**) respectively. Time points are labeled at the bottom. Timepoint 1: pre-heat. Time points 2-9, heat treatment at 35°C or 40°C, including reaching high temperature (0), 0.5, 1, 2, 4, 8, 16, 24 h during heat; time points 10-15, recovery phase after heat treatment, including reaching control temperature (0), 2, 4, 8, 24, 48 h during recovery. Genes involved in gluconeogenesis/glyoxylate cycle were based on MapMan function annotation; genes involved in acetate uptake/assimilation were manually curated based on Durante et. al. (2019) and Johnson et. al. (2013). See the interactive figures with gene IDs and annotations in Supplementary Data 10, gluconeogenesis_glyoxylate cycle.html. More information about genes involved in acetate uptake/assimilation can be seen in Supplementary Data 1 and 2 using the gene IDs on the figure.

Supplementary Fig. 9

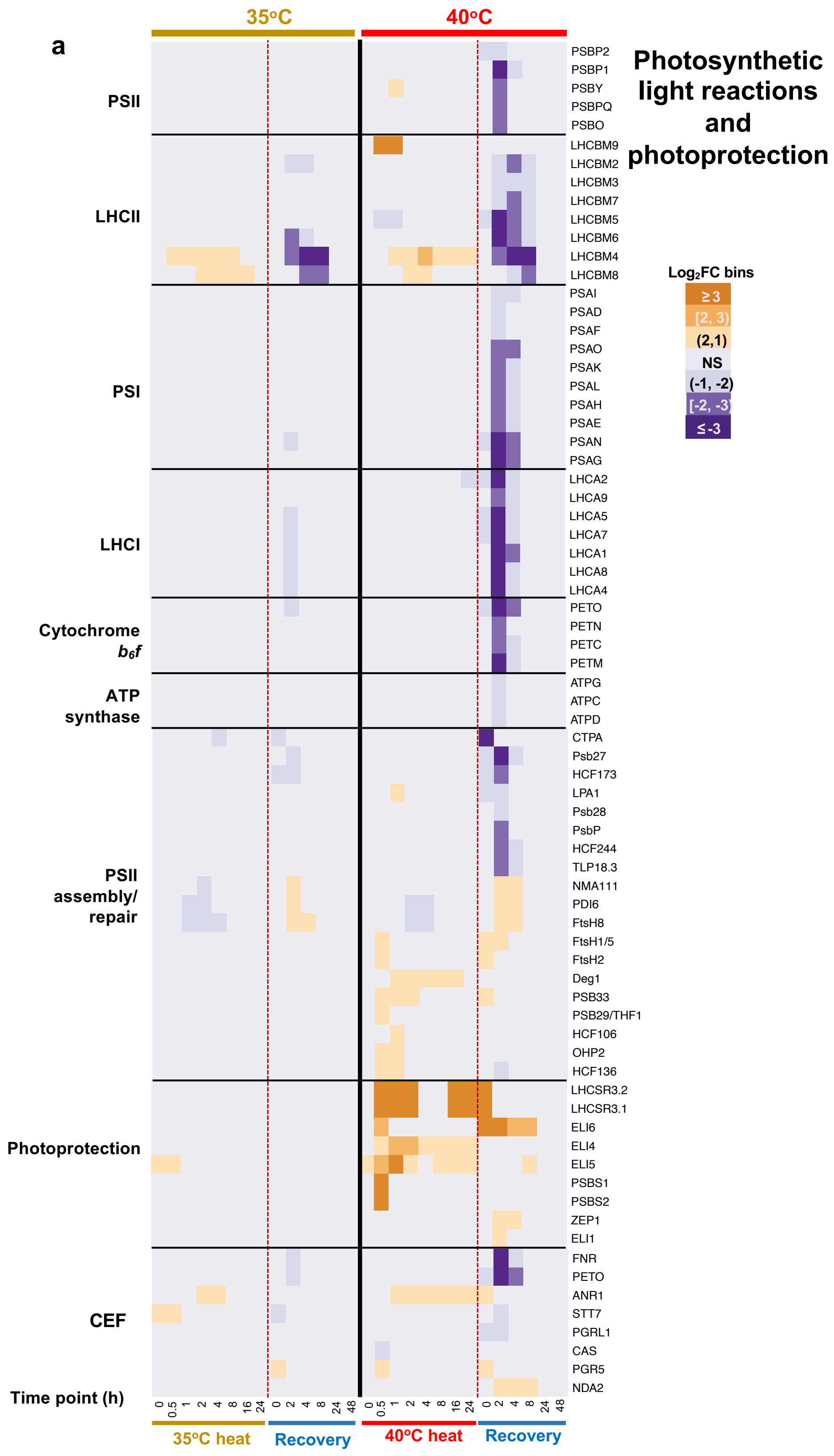

Supplementary Fig. 9

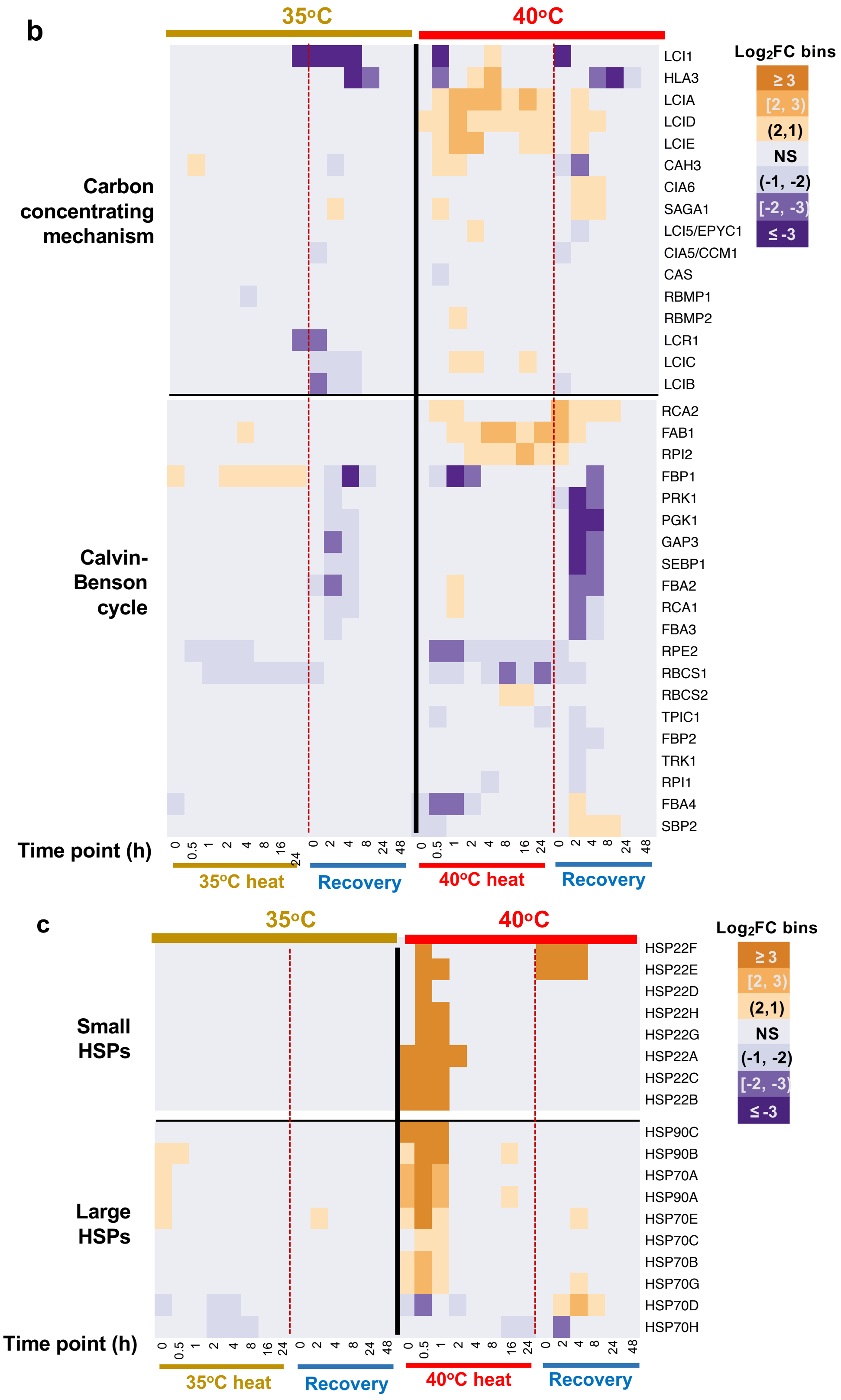

Supplementary Fig. 9

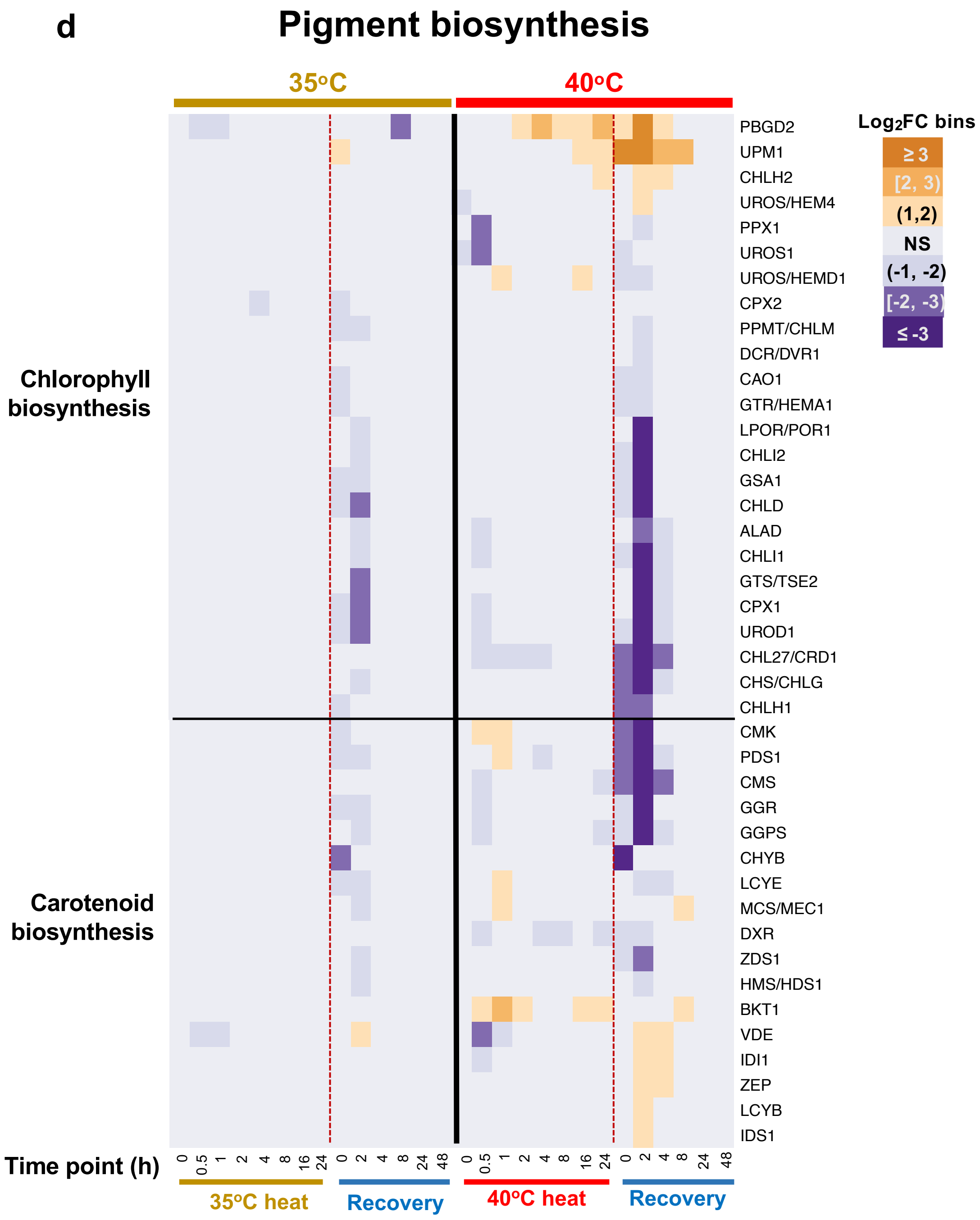

Supplementary Fig. 9

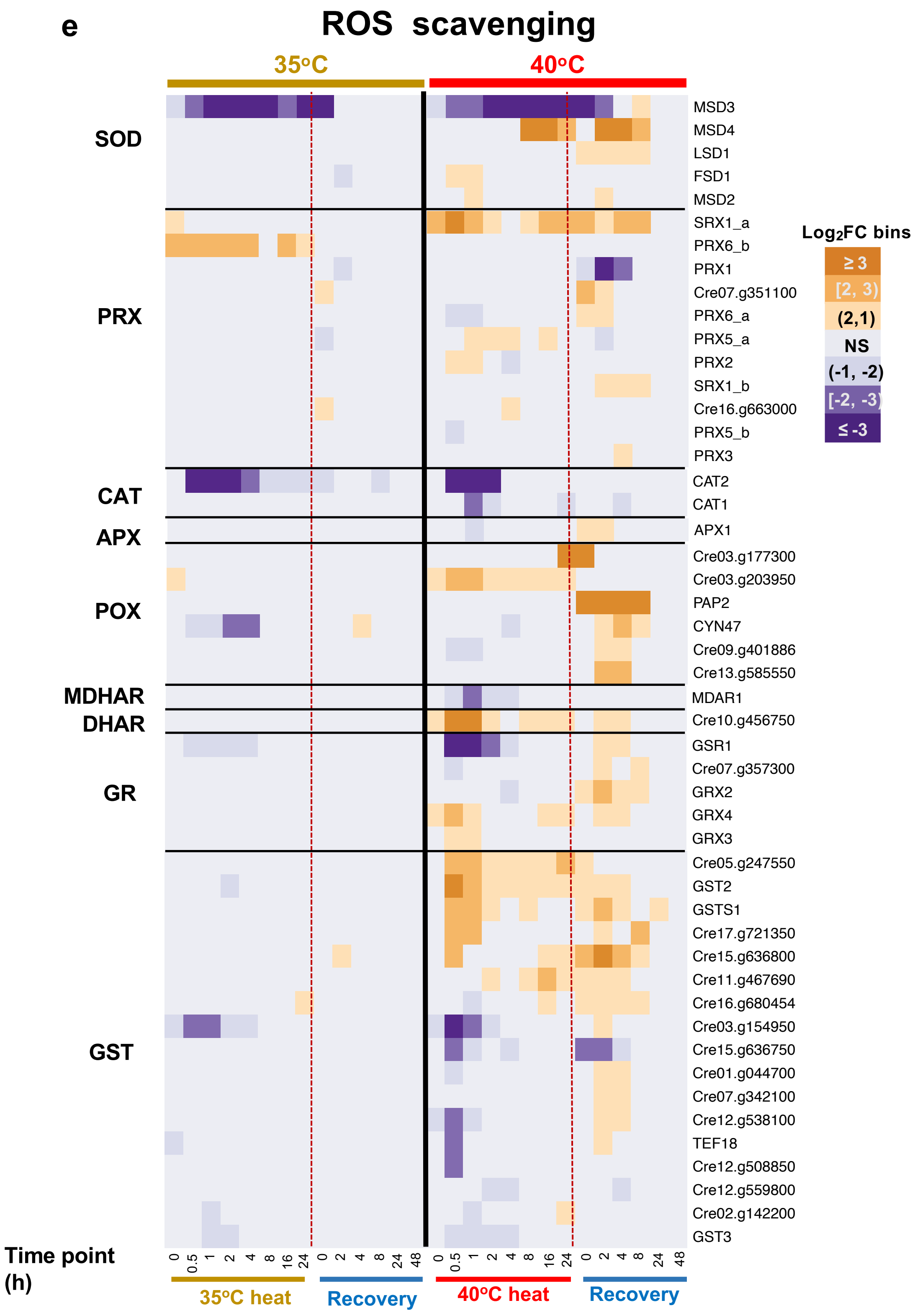

Supplementary Fig. 9

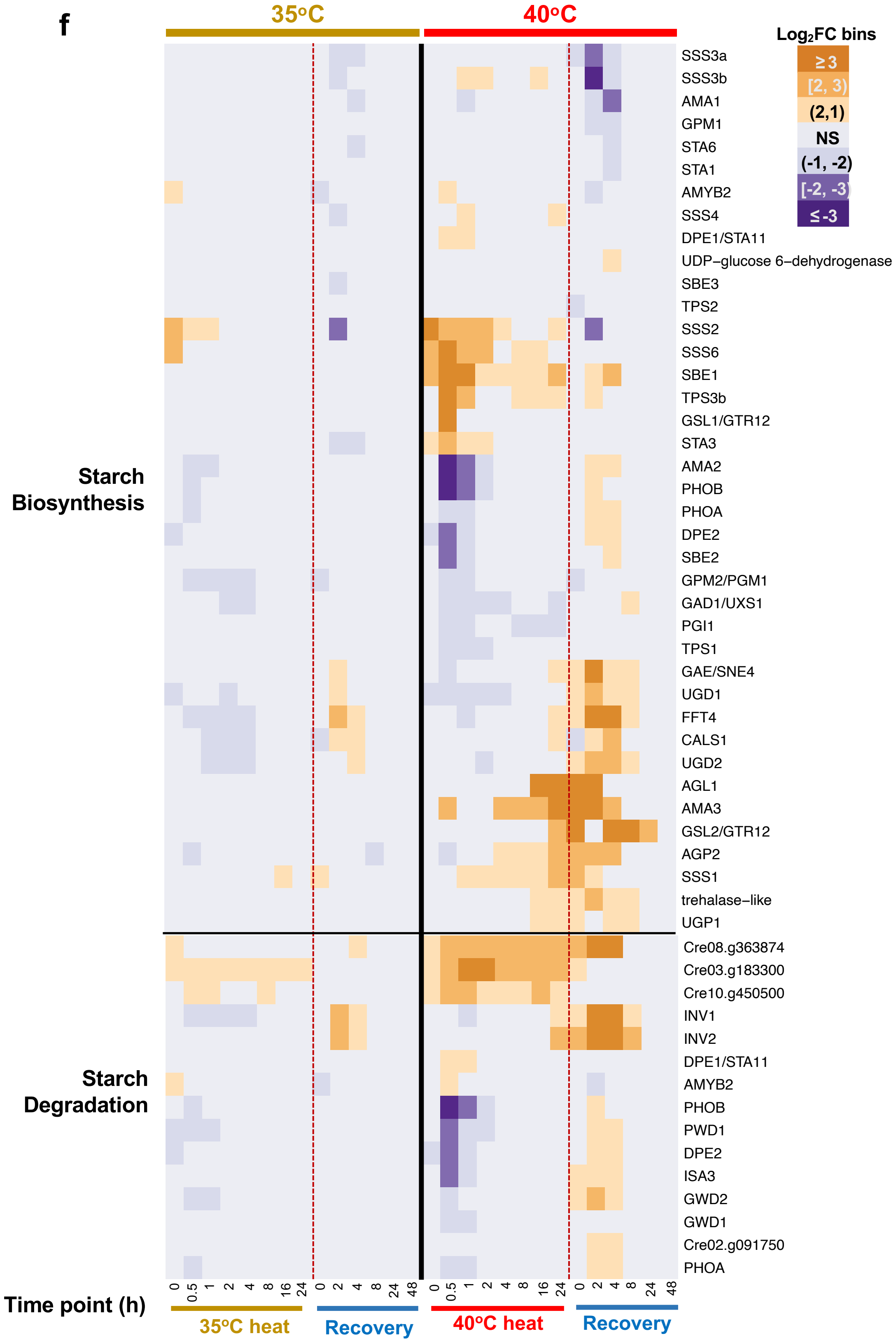

Supplementary Fig. 9

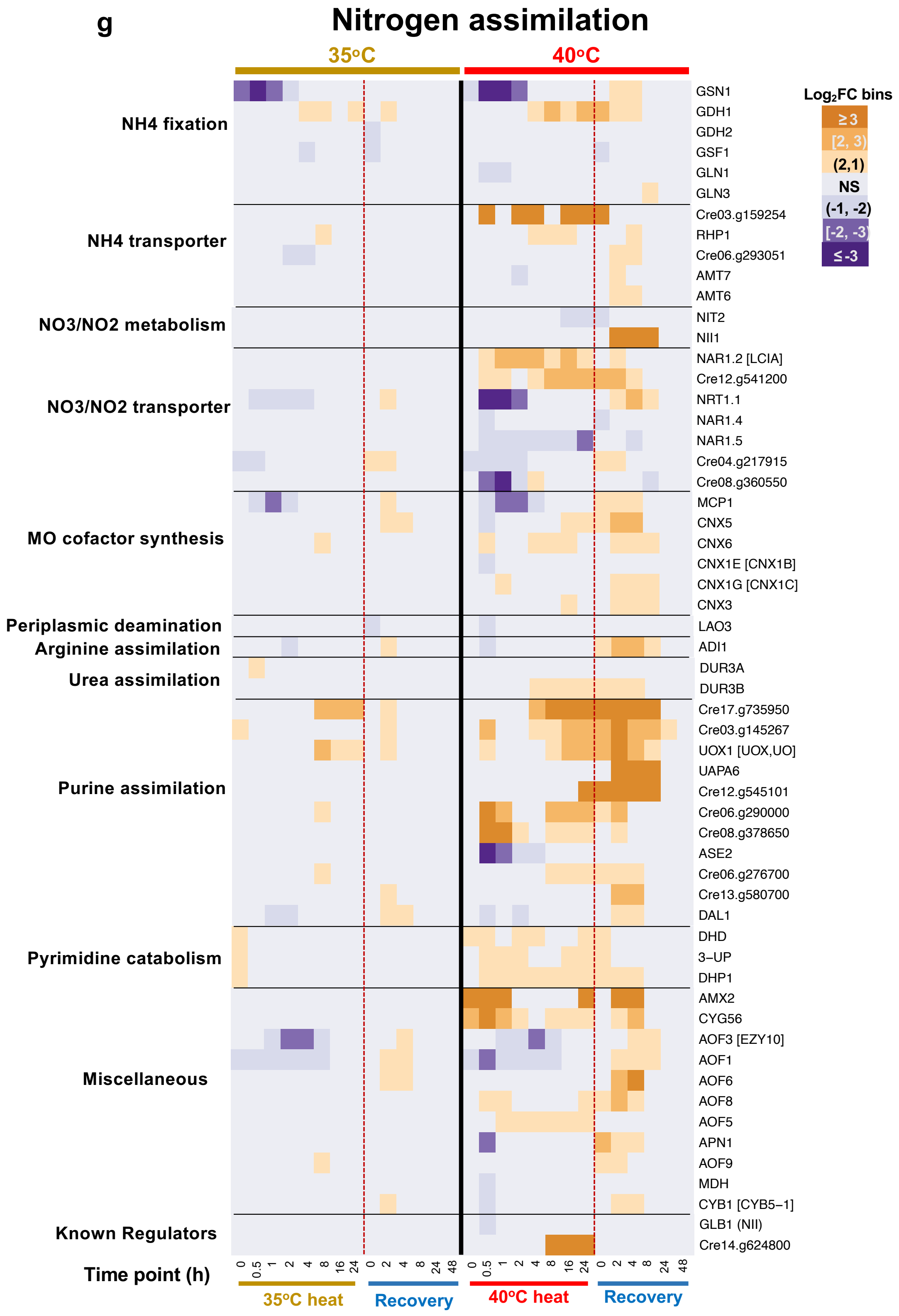

Supplementary Fig. 9

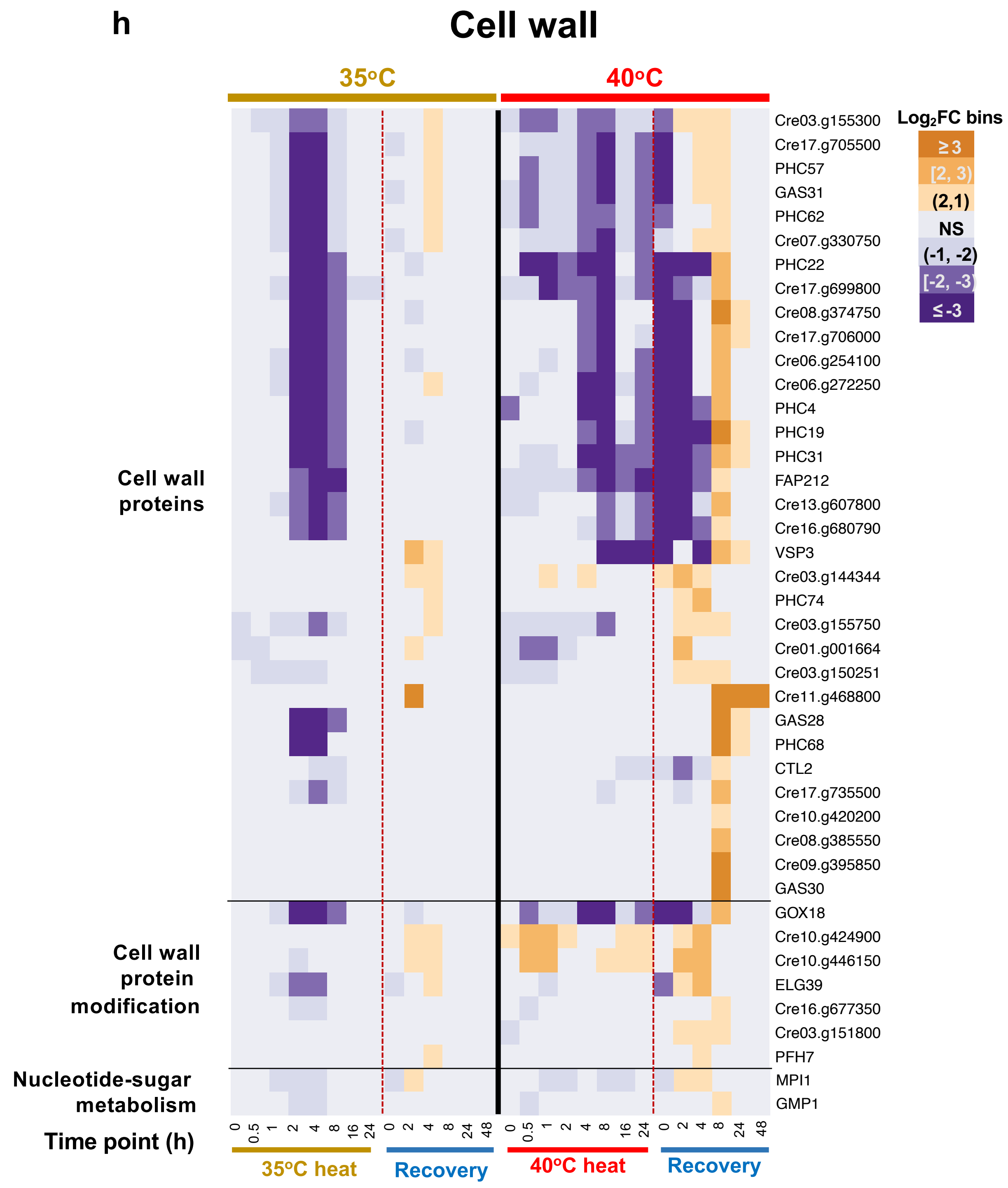

Supplementary Fig. 9

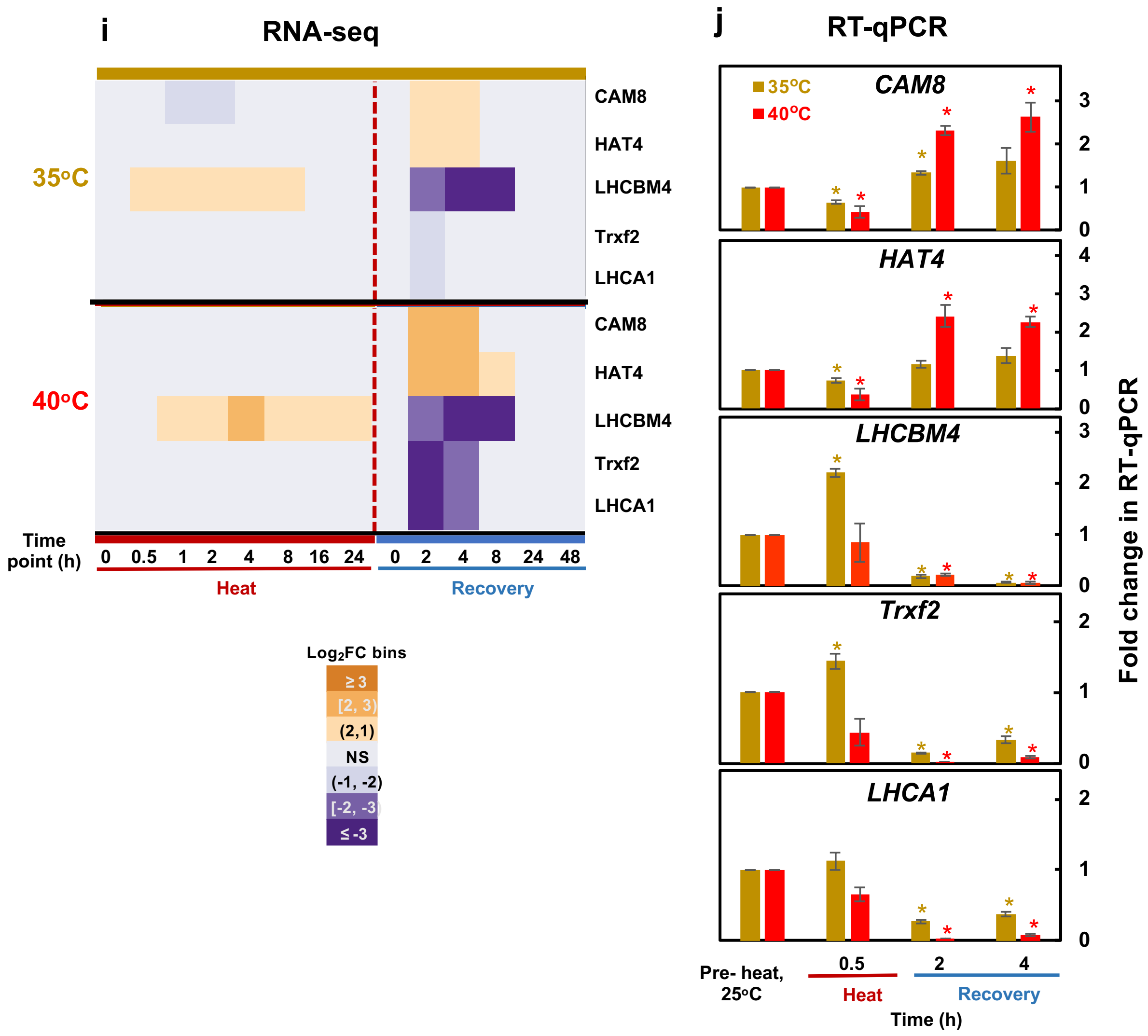

Supplementary Fig. 9: The expression of key pathways was differentially regulated with 35°C or 40°C treatment. Expression patterns of differentially expressed genes in the select pathways are displayed: **(a)** Photosynthetic light reactions and photoprotection, **(b)** carbon concentrating mechanism and Calvin-Benson cycle, **(c)** heat shock proteins (HSPs), **(d)** chlorophyll and carotenoid biosynthesis, **(e)** reactive oxygen species (ROS) scavenging pathways, (**f**) starch biosynthesis/degradation, (**g**) nitrogen assimilation pathways, (**h**) cell wall pathways. (**i**, **j**) RT-qPCR results validated several down-regulated genes related to photosynthetic light reactions (bottom three genes) during the recovery of 2 and 4 h. The top two genes showing opposite expression during recovery are served as controls. *CAM8*, Cre03.g150300, encoding calmodulin-like protein; *HAT4*,

Cre07.g354100, encoding histone acetyltransferase; *LHCBM4*, Cre06.g283950, encoding light-harvesting Chlorophyll a/b binding protein of LHCII; *TRXF2*, Cre05.g243050, encoding thioredoxin f2; *LHCA1*, Cre06.g283050, encoding light-harvesting protein of photosystem I. **(i)** Fold-change heatmap of select genes. Data for 35ºC (top) and 40ºC (bottom) treatment groups were separated by a black horizontal solid line. **(j)** RT-qPCR validation of select genes at the indicated time points. For RT-qPCR results, the fold-changes were calculated by normalizing the relative expression values at different time points with different treatments to the reference gene *CBLP* and the pre-heat time point. Values are mean ± SE, *n* = 3 biological replicates. Statistical analyses were performed with two-tailed t-test assuming unequal variance by comparing treated samples with pre-heat samples (*, $P<0.05$, the color of the asterisks matches the treatment condition). (**a**-**i**) FC, fold-change. Differential expression model output $\log_2$FC values were sorted into different expression bins as follows: highly up-regulated: $\log_2FC \geq 3$; moderately up-regulated: $3 > \log_2FC \geq 2$; slightly up-regulated: $2 > \log_2FC > 1$; NS: not significant; slightly down-regulated: $-2 < \log_2FC < -1$; moderately down-regulated: $-2 \leq \log_2FC < -3$; highly down-regulated: $\log_2FC \leq -3$. Vertical red dashed lines indicate the switch from high temperature to recovery period in each treatment group. Time points were labeled at the bottom. Time points during heat: 0 h, reach high temperature of 35ºC or 40ºC; 0.5 h, heat at 35ºC or 40ºC for 0.5 h, similar names for other time points during heat. Time points during recovery: 0 h, reach control temperature of 25ºC for recovery after heat; 2 h, recovery at 25ºC for 2 h, similar names for other time points during recovery.

Supplementary Fig. 10

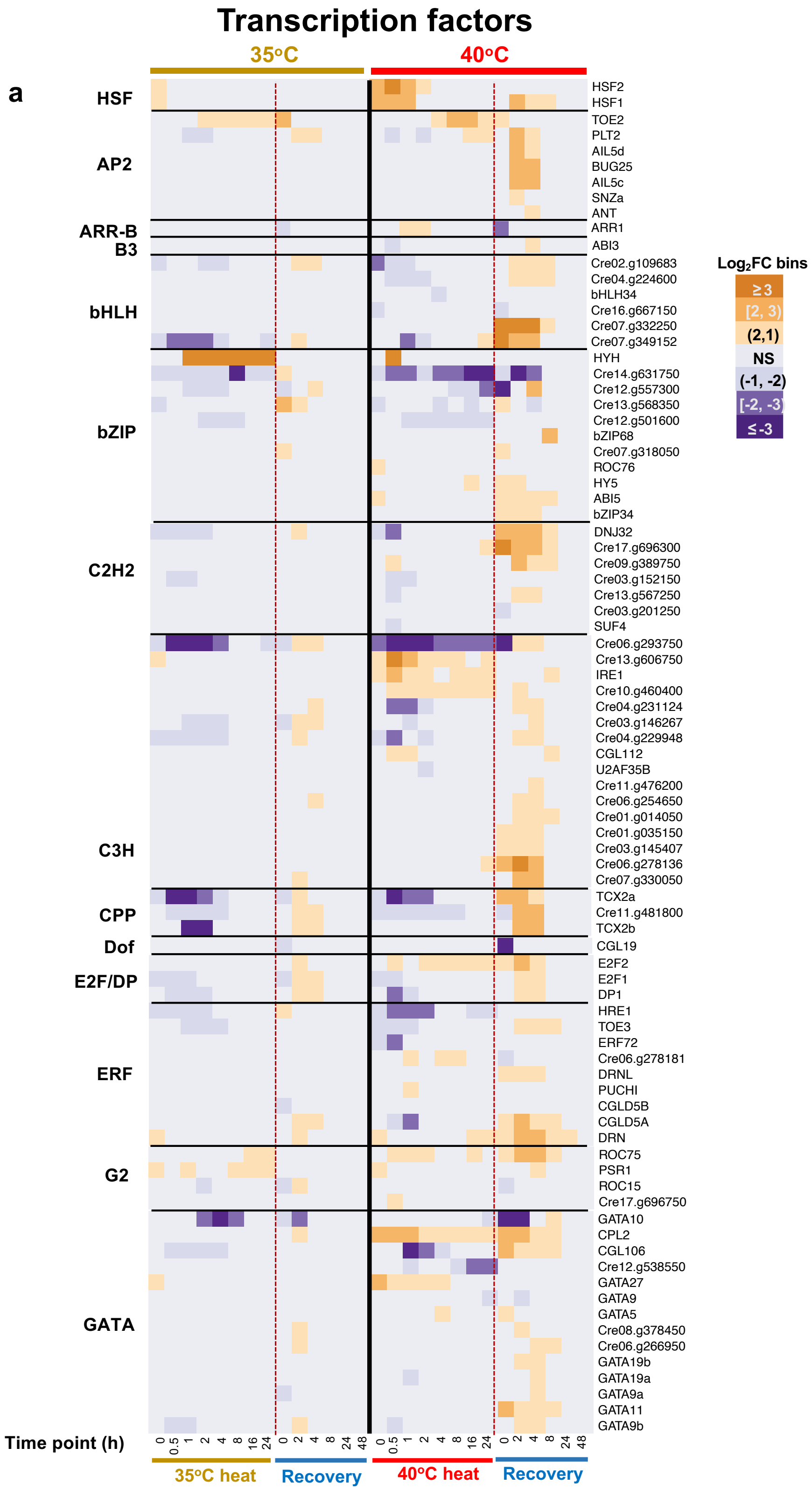

Supplementary Fig. 10

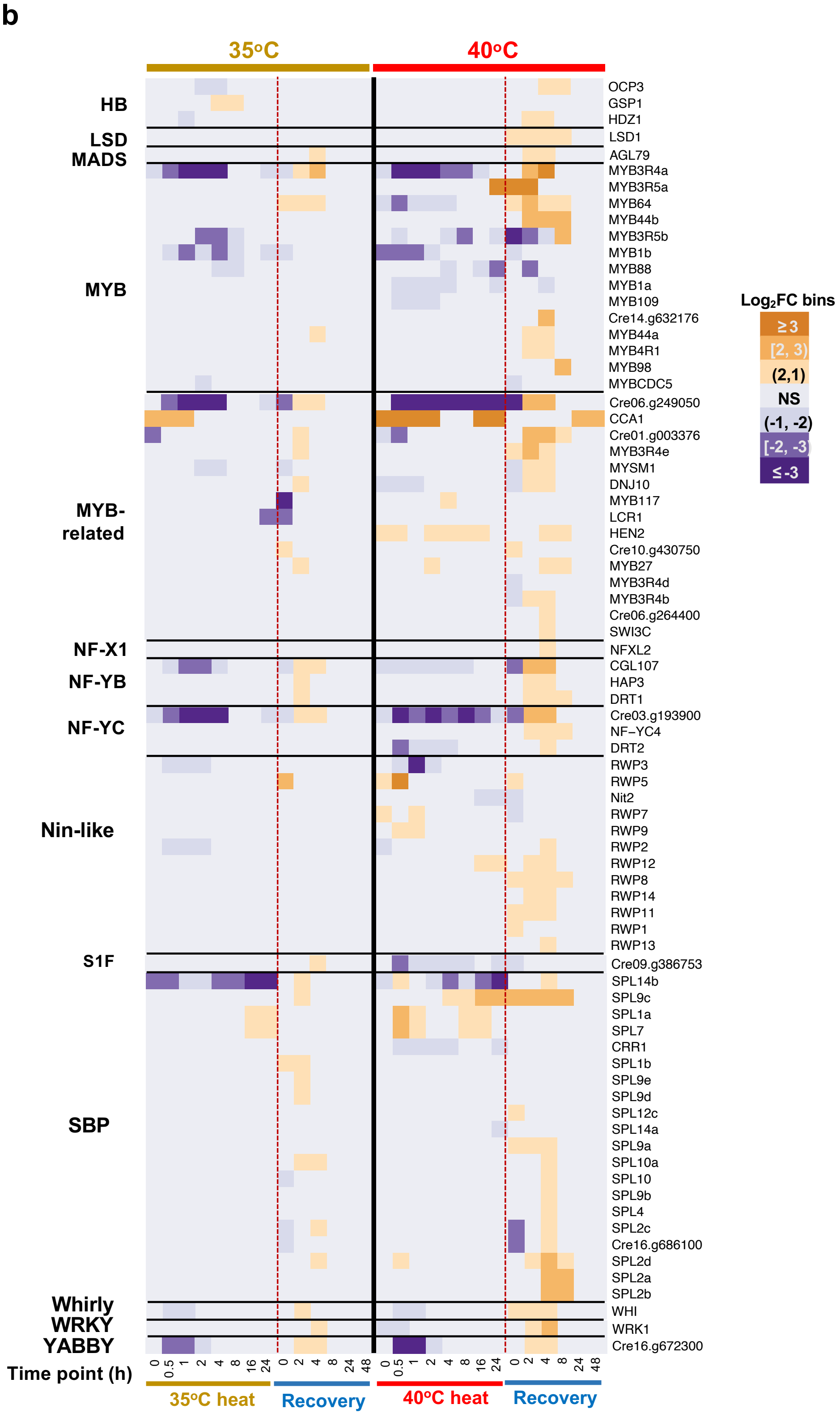

Supplementary Fig. 10: Genes encoding transcription factors had different expression patterns with 35ºC or 40ºC treatment. Expression patterns of differentially expressed genes that encode transcription factors identified using Plant Transcription Factor Database. FC, fold-change. Differential expression model output $\log_2$FC values were sorted into different expression bins as follows: highly up-regulated: $\log_2FC \geq 3$; moderately up-regulated: $3 > \log_2FC \geq 2$; slightly up-regulated: $2 > \log_2FC > 1$; NS: not significant; slightly down-regulated: $-2 < \log_2FC < -1$; moderately down-regulated: $-2 \leq \log_2FC < -3$; highly down-regulated: $\log_2FC \leq -3$. All treatment time points are shown in 35ºC (left) and 40ºC (right), separated by a vertical solid black line. Vertical red dashed lines indicate the switch from high temperature to recovery period in each treatment group. Time points were labeled at the bottom. Time points during heat: 0 h, reach high temperature of 35ºC or 40°C; 0.5 h, heat at 35ºC or 40°C for 0.5 h, similar names for other time points during heat. Time points during recovery: 0 h, reach control temperature of 25°C for recovery after heat; 2 h, recovery at 25°C for 2 h, similar names for other time points during recovery.

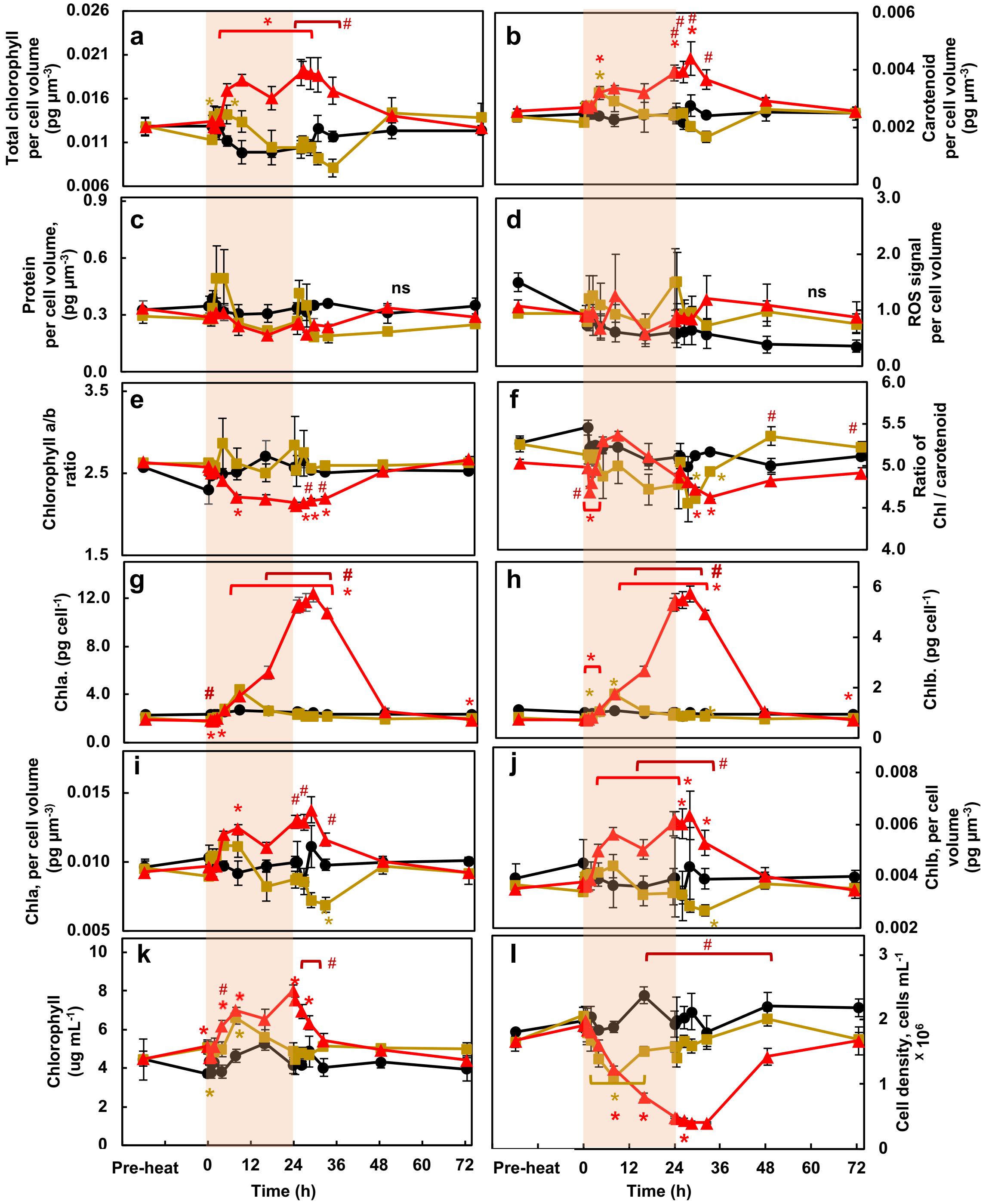

Supplementary Fig. 11. Heat at 40°C increased chlorophyll and carotenoid accumulation per cell volume but not the level of protein or ROS production. (a-d) Chlorophyll (Chl), carotenoid, protein, and reactive oxygen species (ROS) levels normalized to cell volume, respectively; **(e)** Ratios of Chl a and b**; (f)** Ratios of chlorophyll and carotenoid**; (g, h)** Chl a and Chl b per cell; (**i**, **j**) Chl a and Chl b per cell volume; **(k)**

Chlorophyll per mL. (**l**) Cell density in PBR cultures with turbidostatic control. Red shaded areas depict the duration of high temperature. Values are mean ± SE, *n* = 4 biological replicates. Statistical analyses were performed with two-tailed t-test assuming unequal variance by comparing treated samples with 25°C at the same time point (*, $p<0.05$, the colors of asterisks match treatment conditions) or comparing treatments at 35°C and 40°C at the same time point (#, $p<0.05$, the positions of pound signs match the time points). (**c, d**) Not significant, ns.

Supplementary Fig. 12

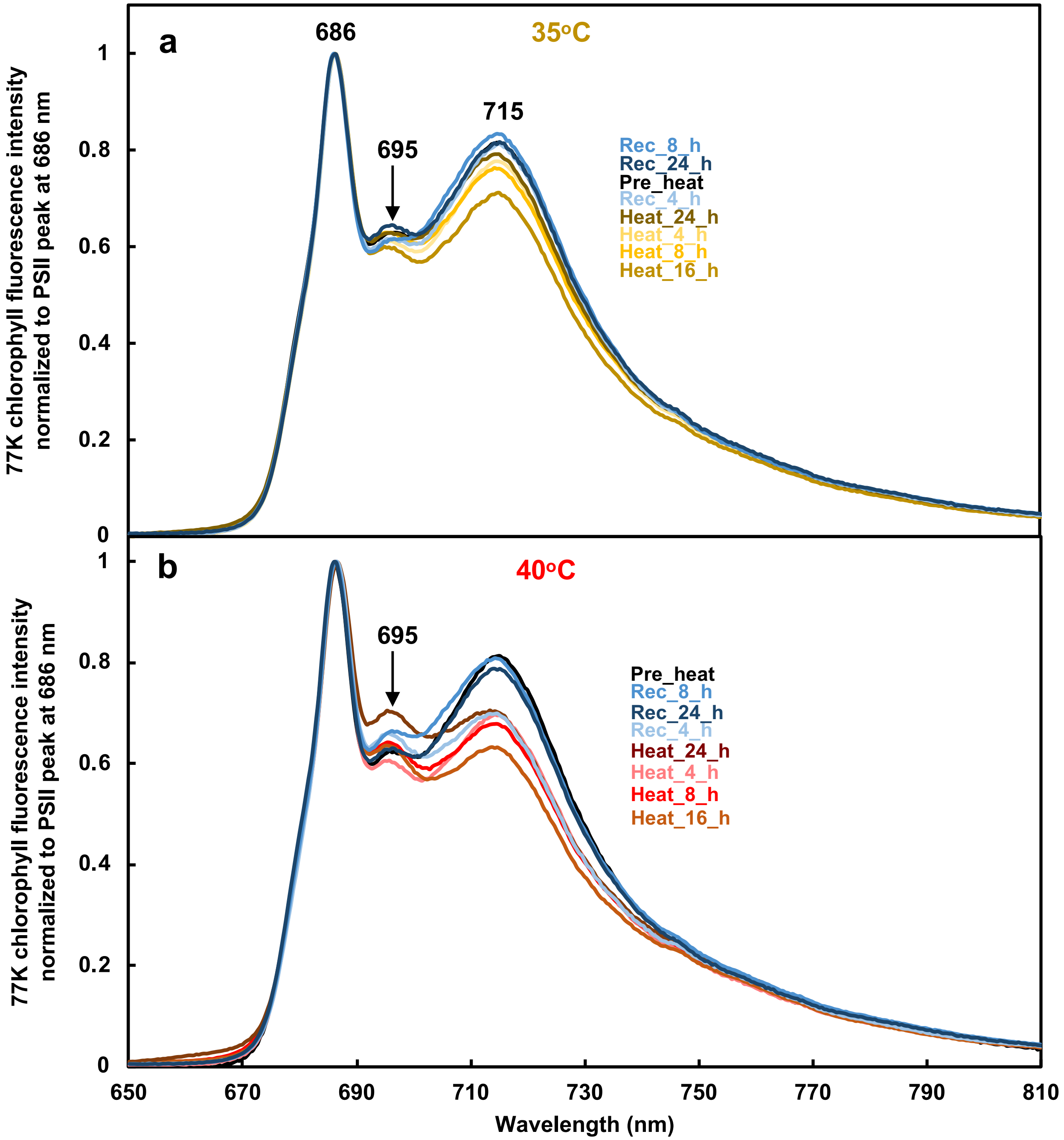

Supplementary Fig. 12. Both 35°C and 40°C heat decreased PSI emission peak around 715 nm but increased PSII emission peak around 695 nm in 77 K chlorophyll fluorescence measurement. 77 K chlorophyll fluorescence spectra from algal cells at different time points before, during and after heat treatments at 35°C or 40°C. Each curve is an average of 3 biological replicates and normalized to PSII peak at 686 nm.

Supplementary Fig. 13

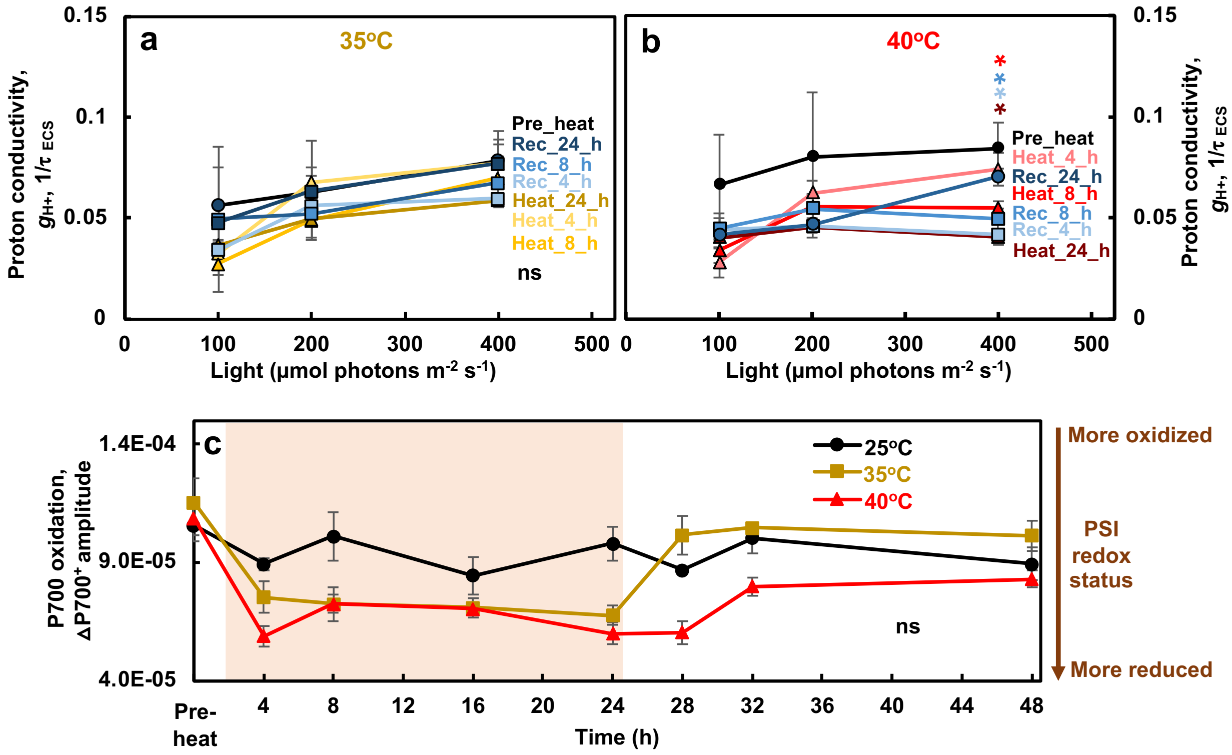

Supplementary Fig. 13. Heat at 35°C and 40°C decreased ATP synthase activity and caused reduced PSI. (a, b) Proton conductivity (g_H^+), proton permeability of the thylakoid membrane and largely dependent on the activity of ATP synthase, inversely proportional to the decay time constant of light-dark transition induced electrochromic shift (ECS) signal (τ_{ECS}); $g_H^+ = 1/\tau_{ECS}$. (**c**) P700 oxidation, measured by the amplitude of absorbance change due to far-red induced oxidized $P700^+$ followed by dark reduction, normalized to chlorophyll contents. Smaller number means smaller amount of oxidizable P700 and more reduced PSI. Red shaded areas depict the duration of high temperature. Mean ± SE, *n*=3 biological replicates. Statistical analyses were performed using two-tailed t-test assuming unequal variance by comparing treated samples with the pre-heat samples under the same light (**a, b**) or constant 25°C samples at the same time point (**c**), or by comparing samples between 35°C and 40°C at the same time point (**c**). P values were corrected by FDR. *, p<0.05, the colors and positions of asterisks match the treatment conditions and time points, respectively. (**a**, **c**) Not significant, ns.

Supplementary Fig. 14

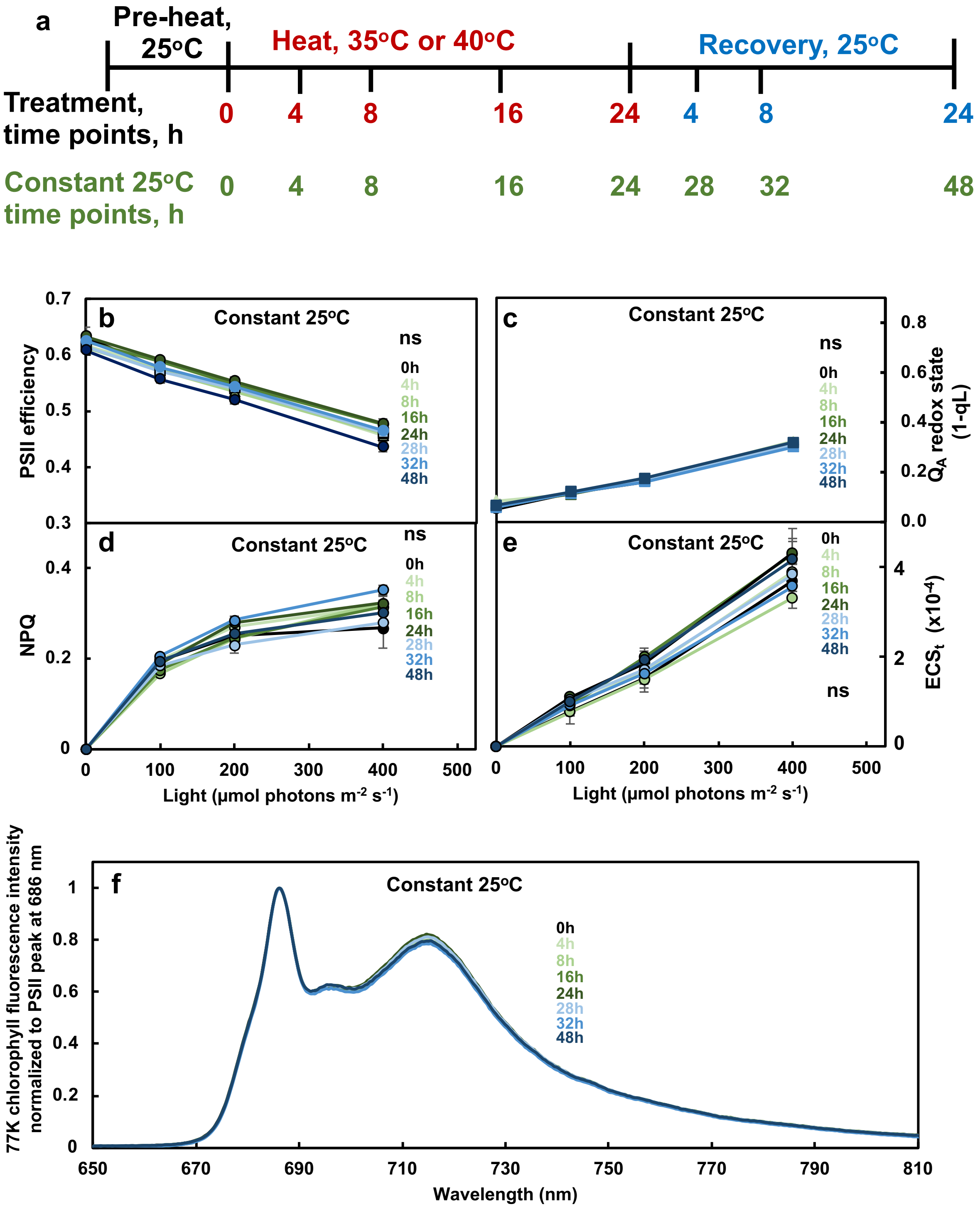

Supplementary Fig. 14. Photosynthetic parameters of algal cultures grown in PBRs with turbidostatic mode were constant without heat treatment. (a) Time points before, during and after heat treatments at 35°C or 40°C and corresponding time points at

constant 25°C. (**b-f**) Photosynthetic parameters from algal cells at different time points during constant 25°C without heat. (**b**) PSII efficiency; (**c**) The Q_A redox state refers to the redox state of chloroplastic quinone A (Q_A), the primary electron acceptor downstream of PSII; (**d**) NPQ (non-photochemical quenching); (**e**) ECS_t, measured by electrochromic shift (ECS), representing the transthylakoid proton motive force, *pmf*. (**b-e**) Values are mean ± SE, *n* = 3 biological replicates. Statistical analyses were performed with two-tailed t-tests assuming unequal variance by comparing different time points with 0-h time point (equivalent to the pre-heat time point for experiments with heat treatments). No significant (ns) differences among different time points without heat. (**f**) 77 K chlorophyll fluorescence spectra. Each curve is an average of 3 biological replicates and normalized to PSII peak at 686 nm.

Supplementary Fig. 15

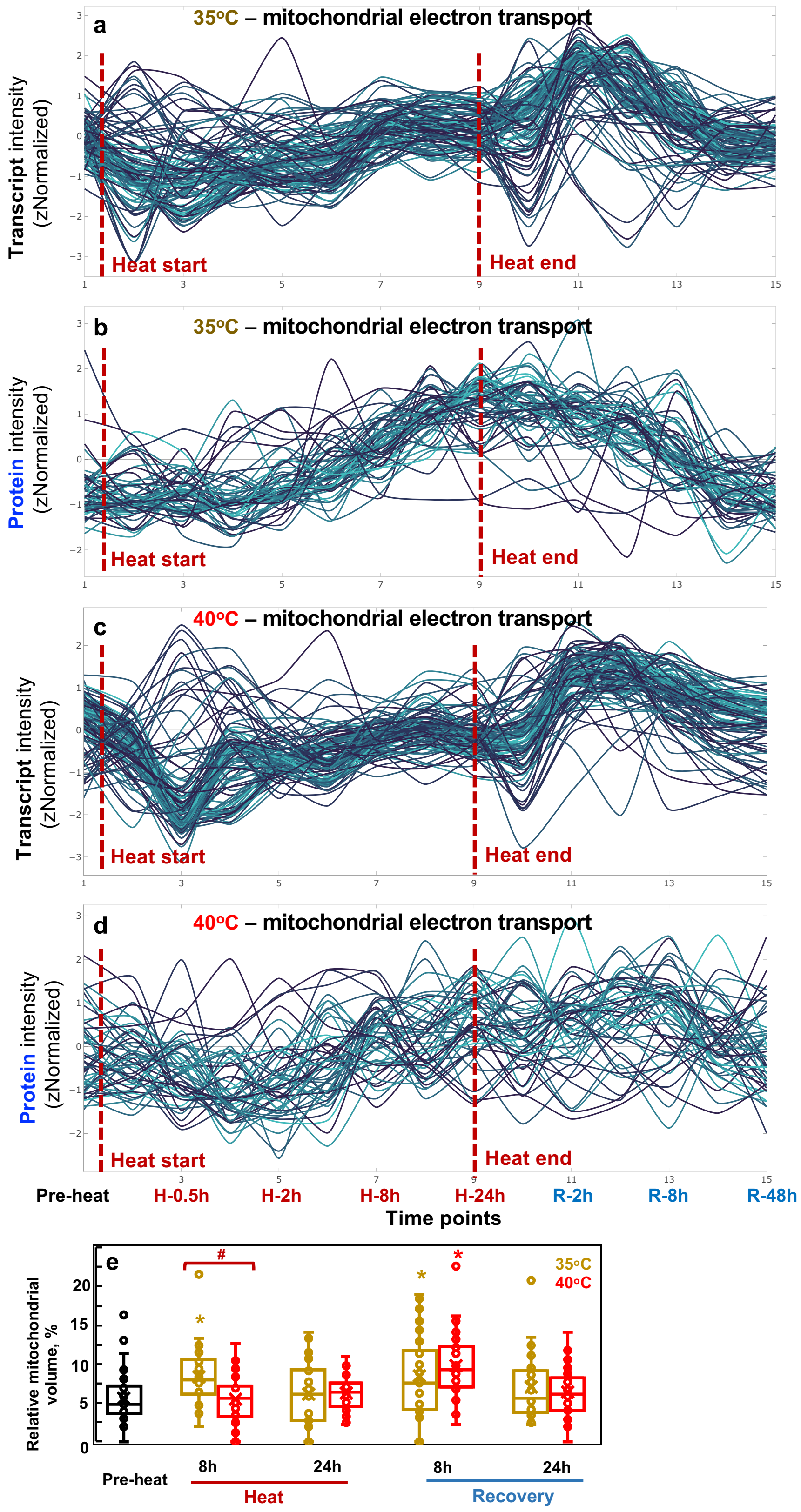

Supplementary Fig. 15. Transcript/protein kinetics and TEM analysis suggested increased and reduced mitochondrial electron transport during 35°C and 40°C heat treatments, respectively. Transcript (**a**, **c**) and protein (**b**, **d**) signals related to the MapMan bin mitochondrial electron transport were standardized to z scores (standardized to zero mean and unit variance) and are plotted against equally spaced time point increments. The red dashed lines indicate the start and end time of heat treatment for 35°C (**a**, **b**) and 40°C (**c**, **d**), respectively. Time points are labeled at the bottom. Timepoint 1: pre-heat. Time points 2-9, heat treatment at 35°C or 40°C, including reaching high temperature (0), 0.5, 1, 2, 4, 8, 16, 24 h during heat; time points 10-15, recovery phase after heat treatment, including reaching control temperature (0), 2, 4, 8, 24, 48 h during recovery. See the interactive figures with gene IDs and annotations in Supplementary Data 10, mitochondrial electron transport _ ATP synthesis.html. (**e**) Relative volume fractions of mitochondria were quantified using TEM images and Stereo Analyzer with Kolmogorov–Smirnov test for statistical analysis compared to the pre-heat condition (*, $p<0.05$, the colors of asterisks match treatment conditions) or between 35°C and 40°C at the same time point (#, $p<0.05$). Each treatment had three biological replicates, total 30 images per treatment.
