# Supplementary material for: Systems-wide analysis revealed shared and unique responses to moderate and acute high temperatures in the green alga Chlamydomonas reinhardtii
Source: Commun Biol. 2022 May 13;5:460. doi: 10.1038/s42003-022-03359-z (PMC9106746; doi:10.1038/s42003-022-03359-z)
Supplement: Supplementary file 3 — Description of Additional Supplementary Files [file 42003_2022_3359_MOESM3_ESM.pdf]

# Description of Additional Supplementary Files

**File name:** Supplementary Data 1
**Description:** Transcriptomics overview. Transcripts per million (TPM) normalized read counts for all genes in all samples; differential expression output data for all genes in all time points; summary of overlapping differentially expressed genes between treatments and time points; genes uniquely up-regulated in 35oC heat; genes more highly differentially expressed in 35oC than 40oC; comparison between this high temperature dataset and the recent work in Arabidopsis (Balfagón et al., 2019); Chlamydomonas heat induced genes with one-to-one orthologous relationship to Arabidopsis genes.

**File name:** Supplementary Data 2
**Description:** Proteomics overview. Normalized protein spectral counts for all proteins identified in each sample, differential accumulation output data for all proteins in all time points, and summary of overlapping differentially accumulated proteins between 35oC and 40oC treatment groups.

**File name:** Supplementary Data 3
**Description:** MapMan functional enrichment for uniquely and overlappingly differentially expressed genes between 35oC and 40oC at each time point.

**File name:** Supplementary Data 4
**Description:** Pearson Correlation Coefficients for transcript-protein pairs in individual MapMan functional categories.

**File name:** Supplementary Data 5
**Description:** WGCNA output, genes belonging to each module and functional enrichment of each module, and summary of genes with unknown functions in each module.

**File name:** Supplementary Data 6
**Description:** Genes used to generate heatmaps for pathways of interest.

**File name:** Supplementary Data 7
**Description:** Proteomics network modeling output, proteins belonging to each module and functional enrichment of each module.

**File name:** Supplementary Data 8
**Description:** Z-scores for transcripts and proteins with 35oC and 40oC treatment and their MapMan functional categories.

**File name:** Supplementary Data 9
**Description:** Transcript and protein correlation plots grouped by MapMan function bins. The heat treatment period (HS) and the recovery period (RE) were split up into three windows each (HS1-3: 0-1 h, 2-8 h, 16-24 h during the heat period; RE1-3: 0-2 h, 4-8 h, 24-48 h during the recovery period after heat treatment, labelled at the bottom of the figure). The average log2(fold changes), lfc, of transcripts and protein pairs in respect to the pre-heat were determined for the three heat (HS, top panels) and three recovery windows (RE, bottom panels), respectively. X and Y, transcript and protein lfc compared with the pre-heat, respectively. All transcript-protein pairs are shown as gray dots. Best fit lines for all transcript-protein pairs are shown in blue and Pearson correlation coefficient are shown on the right in the order for each panel. Transcript-protein pairs belonging to the indicated MapMan functional category (labeled on top of scatterplots) are shown in orange. Each file is .html format and can be opened as an interactive figure with gene IDs and annotations in

a web-browser. The figures can be viewed in detail by clicking on “Show closest data on hover” in the upper right corner of the .html file (Click the upper right corner to see the hidden button).

**File name:** Supplementary Data 10
**Description:** Transcript and protein kinetics grouped by MapMan function bins. Transcript and protein signals related to each MapMan function bin were standardized to z scores (standardized to zero mean and unit variance) and are plotted against equally spaced time point increments as labeled on the x axis. Time points (TP) are labeled at the bottom. Time point 1: pre-heat. Time points 2-9, heat treatment at 35oC or 40oC, including reaching high temperature (0), 0.5, 1, 2, 4, 8, 16, 24 h during heat; time points 10-15, recovery phase after heat treatment, including reaching control temperature (0), 2, 4, 8, 24, 48 h during recovery. Each file is .html format and can be opened as an interactive figure with gene IDs and annotations in a web-browser. The curves can be viewed in detail by clicking on “Show closest data on hover” in the upper right corner of the .html file (Click the upper right corner to see the hidden button).

**File name:** Supplementary Data 11
**Description:** Source data for main figures.
