# Supplementary material for: Systems-wide analysis revealed shared and unique responses to moderate and acute high temperatures in the green alga Chlamydomonas reinhardtii
Source: Commun Biol. 2022 May 13;5:460. doi: 10.1038/s42003-022-03359-z (PMC9106746; doi:10.1038/s42003-022-03359-z)
Supplement: Supplementary file 13 — supplementary_data_10 [file 42003_2022_3359_MOESM13_ESM.zip › 20210726supplementary_dataset10_transcript_protein_kinetics/35/transport.p- and v-ATPases.H+-transporting two-sector ATPase.html]

### 

Transcripts (top): 11  
Proteins (bottom): 7
