# Supplementary material for: Systems-wide analysis revealed shared and unique responses to moderate and acute high temperatures in the green alga Chlamydomonas reinhardtii
Source: Commun Biol. 2022 May 13;5:460. doi: 10.1038/s42003-022-03359-z (PMC9106746; doi:10.1038/s42003-022-03359-z)
Supplement: Supplementary file 12 — supplementary_data_9 [file 42003_2022_3359_MOESM12_ESM.zip › 20210808supplementary_dataset9_transcript_protein_correlation/plots/40/protein.targeting.nucleus.html]

### Panel order

| Position | Window | Condition | Time points |
| --- | --- | --- | --- |
| top left | HS1 | heat treatment | 0 h - 1 h |
| top center | HS2 | heat treatment | 2 h - 8 h |
| top right | HS3 | heat treatment | 16 h - 24 h |
| bottom left | RE1 | recovery | 0 h - 2 h |
| bottom center | RE2 | recovery | 4 h - 8 h |
| bottom right | RE3 | recovery | 24 h - 48 h |
